# High Content Image Analysis Identifies Novel Regulators of Synaptogenesis in a High-Throughput RNAi Screen of Primary Neurons

**DOI:** 10.1371/journal.pone.0091744

**Published:** 2014-03-14

**Authors:** Thomas J. F. Nieland, David J. Logan, Jessica Saulnier, Daniel Lam, Caroline Johnson, David E. Root, Anne E. Carpenter, Bernardo L. Sabatini

**Affiliations:** 1 Stanley Center for Psychiatric Research, Broad Institute of Harvard and MIT, Cambridge, Massachusetts, United States of America; 2 RNAi Platform, Broad Institute of Harvard and MIT, Cambridge, Massachusetts, United States of America; 3 Imaging Platform at the Broad Institute of Harvard and MIT, Cambridge, Massachusetts, United States of America; 4 Howard Hughes Medical Institute, Harvard Medical School, Boston, Massachusetts, United States of America; Georgia Regents University, United States of America

## Abstract

The formation of synapses, the specialized points of chemical communication between neurons, is a highly regulated developmental process fundamental to establishing normal brain circuitry. Perturbations of synapse formation and function causally contribute to human developmental and degenerative neuropsychiatric disorders, such as Alzheimer's disease, intellectual disability, and autism spectrum disorders. Many genes controlling synaptogenesis have been identified, but lack of facile experimental systems has made systematic discovery of regulators of synaptogenesis challenging. Thus, we created a high-throughput platform to study excitatory and inhibitory synapse development in primary neuronal cultures and used a lentiviral RNA interference library to identify novel regulators of synapse formation. This methodology is broadly applicable for high-throughput screening of genes and drugs that may rescue or improve synaptic dysfunction associated with cognitive function and neurological disorders.

## Introduction

The synapse is the subcellular locus of neuronal communication in the central and peripheral nervous system[1]. Many neurodevelopmental and neurodegenerative disorders are hypothesized as diseases of synapse formation or communication. Genes encoding proteins involved in synapse formation and regulation are often mutated in monogenetic autism spectrum disorders and intellectual disabilities[2]–[4].

The complexity of the organization, formation and regulation of the synapse, built of a large and diverse number of scaffold proteins, trans-synaptic adhesion molecules, neurotransmitter transporters, signaling molecules and cytoskeletal proteins[1] necessitates automated high throughput screening methodology to systematically identify the numerous factors controlling synapse structure and function. Traditional single gene studies, although highly successful, cannot fully capitalize on the wealth of proteomic and genomic data now available for the brain. Forward genetic, mainly non-quantitative, screens in fly and worm demonstrate the feasibility and value of such a screening approach, but invertebrate neural systems do not capture all the neural functions associated with cognition and disease in higher order organisms.

Here we present an automated high-throughput screening method of mouse primary cortical neurons coupled with a novel multiplexed image analysis algorithm for unbiased, unsupervised analysis of synapse development. We used this platform for a systematic loss-of-function genomic screen in 96-well microplate format targeting over a hundred endogenous expressed genes using lentiviral delivery of short hairpin RNAs (shRNAs), which permits expression of the RNAi reagent in all neurons in the well. We validate the approach by identifying previously established regulators of synapse function, and extend our understanding of synapse formation by identification of novel regulators. The methodology and image analysis algorithm presented are valuable tools for high-throughput genomic and drug screening approaches that aim to identify genes and drugs that regulate or restore (e.g. in disease models) synapse development of rodent or human embryonic stem cell (ESC) or induced pluripotent stem cell (iPSC) derived neurons.

## Methods

### Content of the shRNA lentiviral library

A pLKO.1 based lentiviral library consisting of 597 unique 21 mer shRNA sequences driven by a U6 promoter[5] was designed to suppress the expression of 118 endogenous mouse genes: 10 adenylate cyclases, 16 phosphodiesterases, 25 dual specificity protein phosphatases, 41 genes involved in G-protein signaling (9 G-protein coupled receptors, 6 G-protein-coupled receptor kinases, 23 regulators of G-protein signaling (RGS family), one scaffold protein, one protein kinase and one gene (LOC100047944) since retracted from Pubmed) and 23 glutamate receptors. One hundred and fifteen genes were targeted by five unique hairpin sequences (four hairpins targeting the coding sequence (CDS), one the 3′ untranslated region (UTR)) and three genes by 4 unique hairpins (three targeting the CDS, one the 3′UTR). We included five unique positive control hairpins targeting the positive control genes Psd95, Synapsin-1, Tsc1, Gephyrin and Pten that are known to be critical for synapse development. We included on each plate unique shRNA negative control sequences targeting GFP, RFP, and luciferase. A GFP expressing vector controlled both virus production by HEK293 cells and infection of primary neurons. The lentiviral vector contains a Pgk1 promoter driven PAC (puromycin N- acetyl-transferase) gene to select infected neurons by conferring resistance to the selection drug and ribosomal inhibitor puromycin, an aminonucleoside antibiotic from *Streptomyces alboniger*. Table S1 presents target sequences of all shRNA reagents tested.

The library consisted of seven 96-well plates, each plate containing approximately 85 spatially randomly distributed shRNAs targeting the mouse genes. All five hairpins targeting the same gene were located on the same 96-well plate. Each plate also contained five different negative (non-targeting) control shRNA lentiviruses that target GFP, RFP, β-galactosidase or luciferase, 2 GFP expressing viruses (as a control for virus production) and 4 empty wells (to confirm puromycin selection). An eighth 96-well negative control plate was built of 80 unique control shRNA expressing lentiviruses targeting GFP, RFP, β-galactosidase and luciferase to create a control null distribution to benchmark the effects of the shRNAs targeting the mouse genes in the screen.

### High-throughput production of shRNA lentiviral library

A third generation lentiviral vector system consisting of three different plasmids encoding (1) *gag*, *pol* and *rev*, (2) VSV-G envelope, (3) pLKO.1-shRNA vector was transiently transfected into HEK293 cells in 96-well plates to produce virus in high-throughput. The lentiviral supernatant consisting of 10% serum and 10% albumin in DMEM medium was quality control checked in Alamar Blue survival A549 cell survival assays for appropriate high (>5E7) titer relative to a standard pLKO.1 reference virus. The limited variability (within a 2 fold-range) of titer across the library was instrumental in obtaining uniform infection conditions across all shRNAs of the library[5] and reproducible perturbations of synapse and dendrite development.

### High-throughput culture of mouse embryonic cortical neurons

Neuronal cultures were prepared by enzymatic (0.01% DNAse (Cat# DN25, Sigma), 10 unit per mL of Papain (Cat# LS 00319, Worthington) digestion of cortices harvested from mouse embryos (embryonic day 18, C57Bl/6 mice) following standard procedures. Neurons were seeded robotically in 100 or 200 μl neurobasal medium containing 2% B27, 1% penicillin/streptopmyces and 2 mM Glutamax (all from Invitrogen) at a density of 15,000 or 20,000 neurons per well (depending on the experiment) in 96-well microtiter plates (black wall, clear bottom; BD Falcon) coated with poly-D-lysine (Sigma). They were grown at 37°C and 5% CO2 in a humidified atmosphere. On day-in- vitro (DIV) 4 or 5 the neurons were infected with the shRNA lentiviral library that ranged between 3 and 10 μl virus, depending on the titer of the library. On DIV6 they were exposed to puromycin (final concentration of 0.2 μg/ml. Cat# P9620, Sigma) to select infected cells, and fixed on DIV14 with a solution of 4% paraformaldehyde, 4% sucrose in phosphate buffered saline (PBS). All liquid handling steps including infection (below) were performed with a JANUS (Perkin Elmer) liquid handling robot.

### High throughput viral infection optimization

Infection conditions were optimized by exposing after 4 days *in vitro* culture (DIV4) primary cortical neurons in 96-well plates to 293T cell supernatant containing virus and subjecting on DIV6 cells to puromycin selection or control medium as described above. We tested a wide (0–15 μl) range of empty vector pLKO.1 control to identify optimal infection conditions in mouse primary neurons, and used these conditions to test the shRNA expressing lentiviral library in a more narrow range (3–7 μl). On DIV14, neurons were fixed and processed for immunofluorescence using antibodies that recognize the neuronal marker NeuN (Cat# MAB377, mouse, Millipore) and secondary donkey anti-mouse-Alexa488 antibodies (Cat# A21202, Invitrogen) (see below). The number of NeuN positive cells in each well was counted using an Acumen LaserScan (TTP labtech). The neurons were also stained with the dendrite marker MAP2 (NB300-213, chicken, Millipore using a secondary donkey-anti-Chicken-DyLight647 antibody from Jackson Laboratories; Cat# 703-495-155) and imaged on an IXM high content microscope (Molecular Devices) using a 4X objective (4X S Fluor, 0.2 N.A, Nikon) for gross inspection of health. This experiment narrowed down the range of library virus tested (between 3 and 10 μl) prior to the actual synapse screen to identify the optimal viral library dose. The infection efficiency of each individual shRNA was expressed as percentage of Hoechst positive cells in the presence and absence of puromycin determined by an Acumen LaserScan (TTP labtech). The primary screen was done in two batches of four unique 96-well virus plates that were each infected in three to five replicates. The infection efficiency of each individual shRNA was confirmed in line for all 8 virus 96-well plates (mean infectivity across all shRNAs of 101± 19%).

### High throughput lentiviral screen of synapse development

The effect of RNAi mediated suppression of expression of the target genes was assessed on DIV14. First, neurons were fixed at room temperature for 15 min with 4% paraformaldehyde and 4% sucrose in phosphate buffered saline (PBS). They were washed twice with PBS, permeabilized for 10 min at room temperature with 0.25% Triton-X100 in PBS, washed twice with PBS and then blocked for 1 h at 37°C with 5% bovine serum albumin (BSA) in PBS. Primary antibody staining was performed overnight at 4°C in the same buffer containing antibodies that recognized PSD95 (Cat# MAB1596. mouse IgG2a, Millipore), Synapsin-1 (Cat# AB1543, rabbit, Millipore), Gephyrin (Cat# 147 011, mouse IgG1, Synaptic Systems) and Map2 (NB300-213, chicken, Millipore). The next day they were washed five times with PBS, incubated for 1 h at room temperature with fluorescently labeled secondary antibodies (Cat# A21135, donkey anti-mouse-IgG2a-Alexa594 Cat# A21206, donkey-anti-Rabbit-Alexa488; Cat# A2140, Adonkey anti-mouse-IgG1a-Alexa 674 [all from Invitrogen]) and donkey-anti-Chicken-AMCA (Cat# 103-155-155, amino-methyl-coumarin-acetate; Jackson Laboratories), and then washed 5 times with PBS. All washing steps were performed with a robotically operated ELK405 micro platewasher (Biotek). The 96-well plates were processed with a robotically operated automated IXM imaging microscope (Molecular Devices) and a 40X objective (40X S ELWD, 0.6 N.A, Nikon). Nine or 12 fields were imaged per well.

### High-throughput image analysis algorithm for synapse development

A synapse development algorithm was developed that tracked 21 parameters by high-content imaging: number, synapse area, synapse eccentricity, synaptic protein expression levels and density of Psd95, Syn1 and Gphn punctae; number and density of excitatory Psd95-Syn1 and inhibitory Gphn-Syn1 punctae. Dendrite length was tracked to identify images with low dendritic outgrowth and to calculate synaptic densities by dividing the number of punctate per unit MAP2 length.

Image analysis was performed using the open-source CellProfiler software[6]. A serial set of image analysis algorithms (an ‘image analysis pipeline’) was constructed to measure features in each of the Gephyrin, Synapsin-1, PSD-95, and MAP2 channels. Each of nine or twelve sites per well was analyzed independently, and the image processing was parallelized by sending batches of images to the Broad Institute's computing cluster. The analyzed data was merged and stored (http://www.cellprofiler.org, Broad Institute) in a MySQL (Oracle, Inc.) database.

The CellProfiler pipeline will be included in Supplementary Materials with further details and made freely available online (http://www.cellprofiler.org/published_pipelines.shtml) but is briefly outlined here. First, an illumination correction function was calculated for each channel to correct for persistent illumination variations across each 96-well plate (due to many possible sources, including optical hardware irregularities, illumination patterns, or shading). Illumination functions were created by averaging all the images of each channel across each plate, then smoothing the resulting image with a large median filter (100×100 pixels), and finally saved offline. Each site was then processed in a separate processing pipeline as follows. The four channels' raw images were divided by their respective plate/channel illumination function. Next, translational misalignments were corrected by maximizing the mutual information of the PSD-95 and Gephyrin channels relative to the Synapsin-1 channel. The three synapse channels were enhanced using a top-hat morphological filter with a disk structuring element from 6–8 pixels in diameter. The MAP2 dendrite channel was enhanced using an ImageJ plugin[7] executed within CellProfiler called Tubeness[8] http://www.longair.net/edinburgh/imagej/tubeness/). Summed dendritic arbor lengths per image were measured by image segmentation[9] and morphological skeletonization with despurring. Synaptic objects were segmented and low intensity objects were filtered based on thresholds determined empirically for each experimental batch. Overlap of each synaptic object pair was measured as pixel area overlap as well as a simple count of objects with any regional overlap. Synaptic densities were also calculated (number of synapses per μm MAP2 length). The effect of shRNAs on synaptogenesis and dendrite development was assessed by first aggregating the data of images taken within the same well and then over replicate wells.

### shRNA knockdown validation by western blot

Neuronal cultures were prepared from cortices that were harvested from mouse embryos (embryonic day 18, C57Bl/6 mice), seeded in 12well poly-D-lysine coated plates (BD Falcon) at a density of 400,000 cells/well and cultured as described for 96-well format. On DIV4, half the medium was removed and conserved. In contrast to 96w- plate infections, optimal infection of these larger scale neuronal cultures required application of the cationic peptide protamine sulfate (6 μg/ml, MP Biomedicals), which neutralizes the charge repulsion between the lentiviral virions and sialic acid residues on the cellular surface and centrifugation at room temperature for 30 min, 2200 g. The cells were placed in the tissue culture incubator for 3–5 hr (37°C, 5% CO2 in a humidified atmosphere), after which the protamine sulfate containing supernatant was removed and replaced with the conserved medium. Neuronal cultures were placed back in to the tissue culture incubator and subjected to puromycin selection on DIV6. On DIV14 the culture medium was removed and cell lysates prepared and analyzed by SDS-PAGE and western blotting using anti-Axin1 (A0481; Sigma-Aldrich) and anti-Grin2C (NB300-107; Novus) antibodies. Expression levels relative to B-actin were determined by measuring the intensity of relevant bands on scanned western blot quantified using LabWorks software (UVP). As control we used a pool of 10 negative control shRNA viruses identified from the negative control plate that had shown to be innocuous in our synapse assay and 293T cell lysates expressing human Axin1 and Grin2C constructs.

### In utero DNA electroporation

All the procedures for animal surgery and maintenance were performed following animal protocols approved by the Harvard Standing Committee on Animal Care and National Institutes of Health guidelines. To target neocortical layer 2/3 pyramidal neurons, E15.5 timed-pregnant female C57BL/6 mice (Charles River, Massachusetts, United States) were anesthetized by 2% isoflurane. Uterine horns were carefully exposed and 1∼2 μl of DNA at the concentration of 1 μg/μl was injected into the lateral ventricle of the left hemisphere of intrauterine embryos using a ∼30–50-μm-diameter pipette sharply beveled at 15°–20° (Narishige, Japan), visually confirming the proper site of correct injection by mixing 0.005% fast green mixed with the DNA. We used the same shRNA pLKO.1 vector that was used to create lentivirus that was used for all other assays and an eGFP-N1 expressing vector (Clontech) as negative control. After injection, the embryo head was held between the paddles of a forcep-type platinum electrode (0.5 mm diameter) and electric pulses were delivered five times per second (50 V, 50 ms) (CUY21 electroporator, NEPA GENE, Japan). Warm PBS was dropped onto embryos periodically to prevent drying. The uterus was placed back into the pregnant mouse, and the anterior muscle and the skin were sutured separately. Pups were housed with the dam until they were needed.

### Spine imaging in acute brain slices

C57BL/6 mice (16–19 days old) were deeply anesthetized with isoflurane and decapitated. The brain was rapidly removed and placed in chilled choline-based cutting solution containing (in mM) 25 NaHCO_3_, 1.25 NaH_2_PO_4_, 2.5 KCl, 7 MgCl_2_, 25 glucose, 1 CaCl2, 110 choline chloride, 11.6 ascorbic acid, and 3.1 pyruvic acid. Coronal sections of the brain were cut at 300 µm thickness using a Leica VT1000S vibratome (Leica Instruments, Nussloch, Germany) in cold cutting solution. Slices were transferred to ACSF containing (in mM) 127 NaCl, 2.5 KCl, 25 NaHCO_3_, 1.25 NaH_2_PO_4_, 2 CaCl_2_, 1 MgCl_2_, and 25 glucose. Both cutting and ACSF solution were saturated with 95% O_2_ and 5% CO_2_ (pH 7.4). GFP expressing Layer 2/3 pyramidal neurons were located in acute brain slices under wide field epifluorescence. Subsequently Z-stacks (1 micron inter-slice spacing) of apical dendrites were acquired as described previously using 2-photon laser scanning microscopy and analyzed using custom software[10]. For each image stack, the number of spines was counted and the total dendrite length measured to calculate the spine density. In addition, the length of each spine was measured to produce cumulative distributions of this parameter for neurons of each genotype.

### Statistical analysis

*Two-tailed t*-test and one way analysis of variance (one way ANOVA) statistical analysis were performed using GraphPad Prism version 6.

### Ethics Statement

Animals were handled according to protocols approved by the Harvard Standing Committee on Animal Care, in accordance with NIH guidelines (Protocol number 03551).

## Results

Functional analysis of synaptogenesis of primary neurons requires consistency of culture conditions, uniform genetic and chemical perturbations or unsupervised automated analysis methodology. Here we describe the development and implementation of lentiviral high throughput RNAi screening methodology and quantitative image analysis algorithms for robust interrogation of the regulatory function of genes in excitatory and inhibitory synaptogenesis of mouse primary cortical neurons (Fig. S1).

### Design of an automated synapse development imaging algorithm

We used the freely available CellProfiler software[6] to develop an automated image analysis pipeline of synaptogenesis. It takes advantage of multiple fluorescence channels and high information content available in images acquired with fluorescence light microscopy. A critical feature of this imaging algorithm is the ability to selectively parse molecular aspects underlying synapse development: (1) synapse morphology (synapse area, eccentricity), (2) synaptic protein expression levels (measured as fluorescence intensity normalized to the area of each individual synapse), (3) the number and density (relative to density length) of individual synaptic punctae in the culture, (4) the molecular composition of the synapse by simultaneously marking different synaptic proteins with different antibodies in a multiplex immunostaining protocol and (5) dendrite outgrowth to calculate synaptic density and determine gross changes in neural development upon RNAi perturbations.

### High-throughput synapse screen

We applied the image algorithm to examine by RNAi the regulatory role of genes in the development of (overlapping) pre-synaptic and post-synaptic boutons of excitatory and inhibitory synapses, visualized with antibodies recognizing Synapsin-1 (Syn1; general pre-synaptic marker), Psd95 (excitatory postsynaptic marker) and Gephyrin (Gphn; inhibitory postsynaptic marker). Excitatory and inhibitory synapses were defined by the presences of overlapping Psd95-Syn1 and Gphn-Syn1 punctae (Fig. S2, S3, S4).

**Figure 1:**
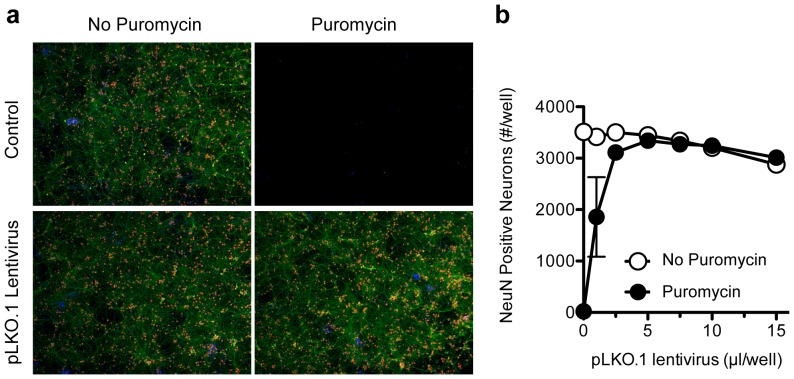
Lentiviral infection optimization of mouse primary cortical cultures in 96- well plate format. (A) Embryonic age 18 cortical neurons were seeded in 96-well plates (DIV0) and mock infected or infected with pLKO.1 empty vector lentivirus (DIV4), subjected to control medium (A,C) or puromycin containing selection medium (DIV6), fixed and processed for immunostaining for NeuN (in red) and Map2 expression (in green) (DIV14). (B) The infection efficiency was assessed by counting the number of NeuN positive neurons in the absence and presence of puromycin using laserscan cytometry.

We identified conditions in which 100% of embryonic day 18 cortical neurons cells are lentivirally infected and perturbed by RNA interference treatment without evidence of cell loss (Fig. 1). This protocol was employed to screen in 96-well plate format (Fig. S1) an RNAi library consisting of 607 individual shRNA sequences targeting mouse genes, some with suspected functions in synapse development (Table S1). Hairpins (shGFP, shRFP, shβ-galactosidase, shLuciferase) that target proteins not expressed in neurons were used as negative controls.

**Figure 2:**
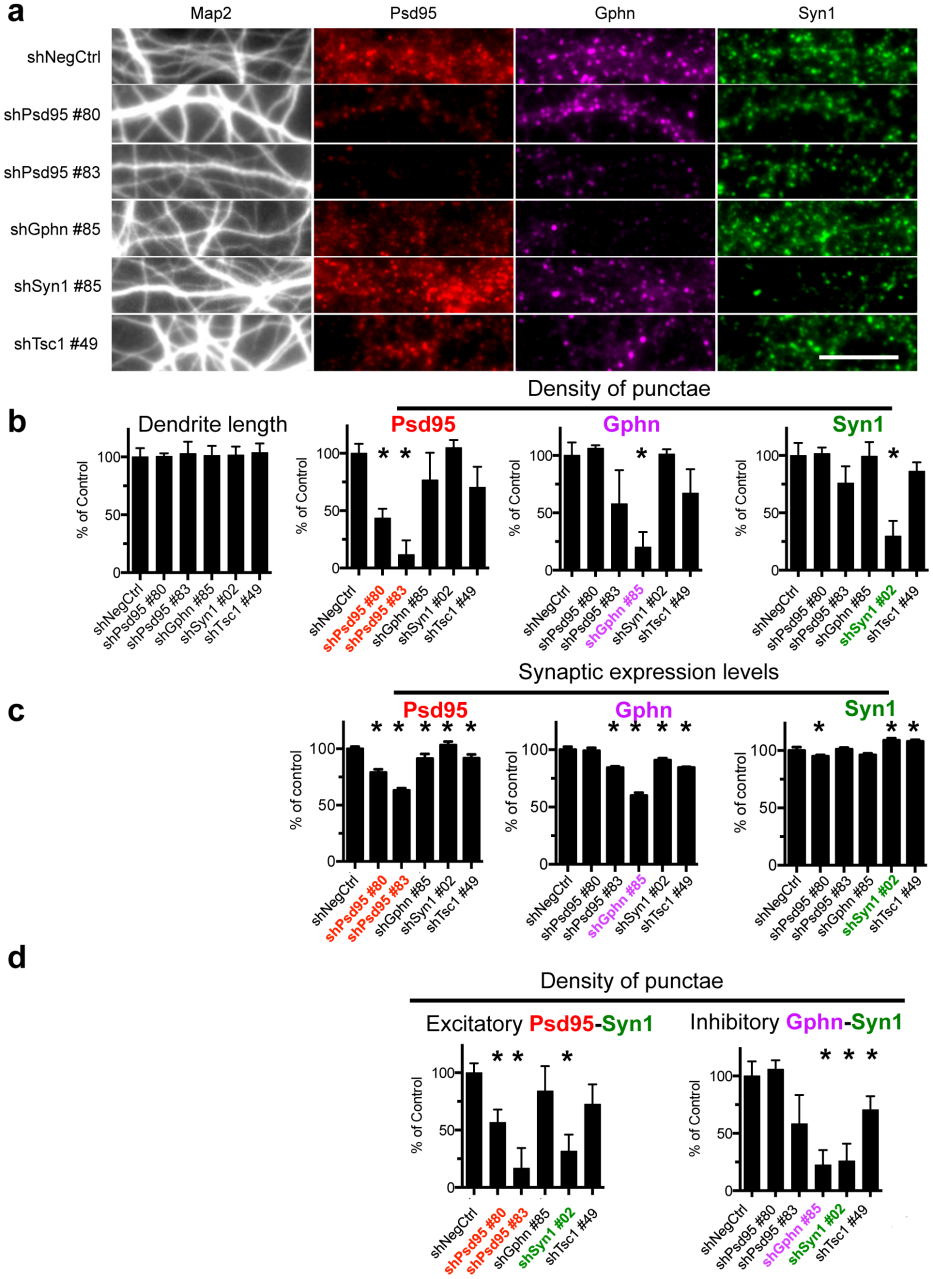
Automated image analysis algorithm quantifies changes in synapses development. (A) Relative to the negative control hairpin (shLacZ #30 targeting B-galactosidase) individual hairpin sequences targeting *Psd95* (two unique sequences), *Gphn*, *Syn1* and *Tsc1* show the expected decrease in development of Psd95, Gphn and Syn1 punctae respectively. Scale bar represents 10 μm. Image results were quantified using the synapse imaging algorithm, showing the effect on (B) dendrite outgrowth and density of individual synaptic punctae and (C) Psd95, Gphn and Syn1 protein expression in the synaptic punctae (per synapse median fluorescence intensity normalized to the area). (D) shRNA targeting Psd95 and Syn1 and shRNA targeting Gphn and Syn1 also reduce excitatory and inhibitory synapse development, respectively. The data in (B) and (D) represent the median and median absolute deviations of the primary screen and 2 independent secondary experiments * p<0.05 or less (2-tailed T-test) compared to respective negative control shRNA. The data in (C) present the mean and standard deviation of the primary screen. The quantitative results and full statistical analysis of all 5 shRNAs targeting each gene is presented in Fig. S5 and Tables S2 and S3.

Positive control shRNAs targeting mRNAs encoded by *Gphn*, *Syn1*, *Psd95* and *Tuberous scleroris-1* (*Tsc1*) included in the screen validated the synapse algorithm. They quantitatively reduced, as expected, the number, density and synaptic expression of their intended Gphn, Syn1 and Psd95 targets (Fig. 2B,C), Fig. S5, statistical analysis in Tables S2 (2-tailed t-test) and S3 (one way analysis of variance (one way ANOVA)), confirming the clear qualitative visual impression of decreased punctae number and Psd95 and Gphn synaptic protein expression levels (Fig. 2A, Fig. S2, S3, S4). They also lowered the density of excitatory and inhibitory synapses, defined by the algorithm as overlapping Psd95 and Synapsin-1 punctae or Gphn and Syn1 punctae, respectively (Fig. 2D, Fig. S5E-F). Strong hairpins also reduced punctae development of the opposite class (e.g. shPSD95 reduces Gphn and Syn1 development), which might be attributable to off-target effects or secondary homeostatic effects induced by changes in network activity.

The optimal infection efficiency (mean infectivity of 101%± 19%) of all 607 shRNAs tested and the little variation in replicate measurements of 21 synapse and dendrite parameters across replicate shRNA perturbations within the primary screen (median %CV of < 7%) and between the primary screen and two independent secondary screens (7% < median %CV < 25%) (Fig. S6) demonstrated the reproducibility and robustness of the RNAi screening and imaging methodology.

**Figure 3:**
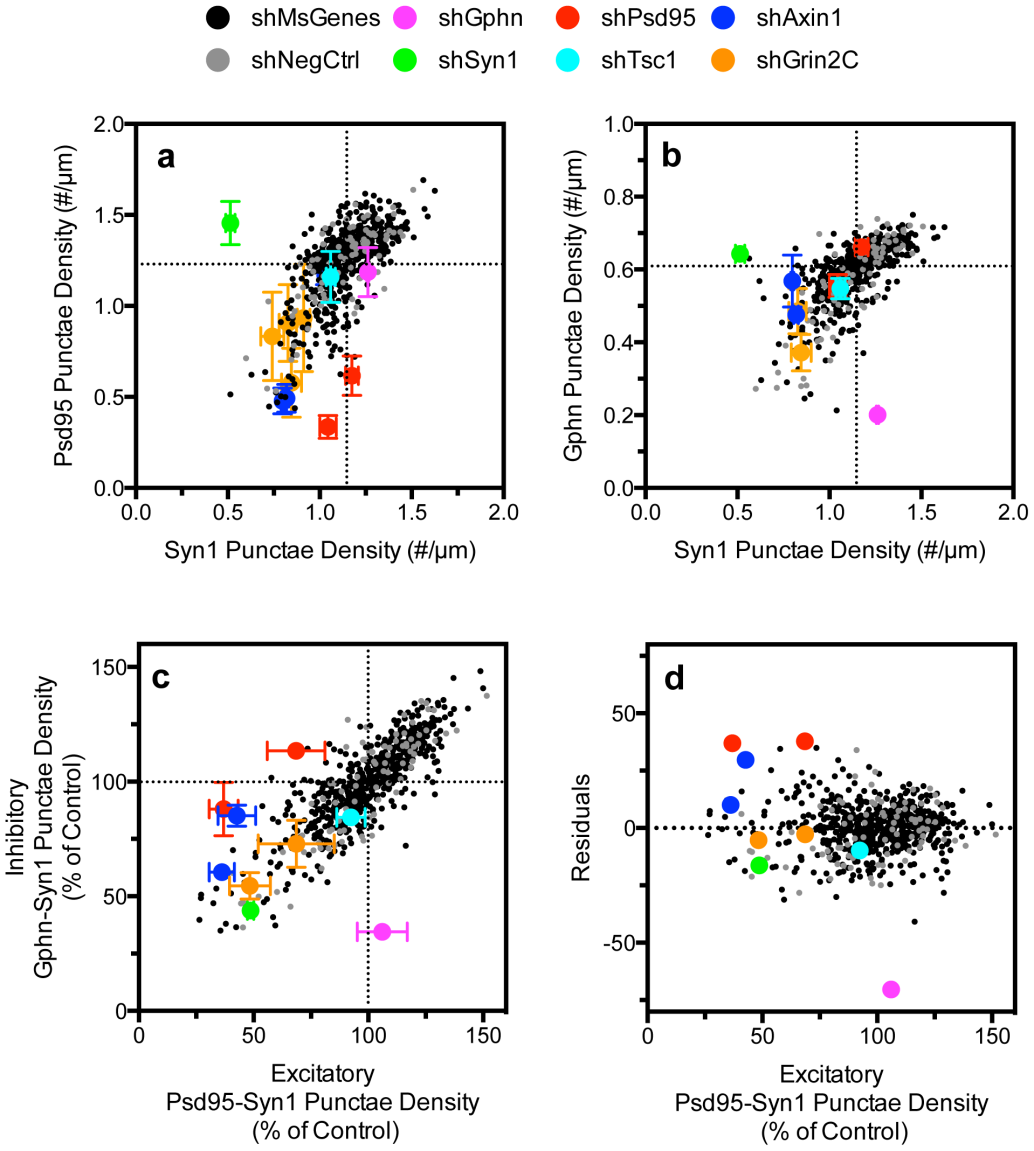
Primary screen analysis of the effect of shRNAs on synapse development. The effect of 607 unique shRNAs targeting mouse genes and of the negative control hairpins correlated well between (A) post-synaptic Psd95 and pre-synaptic Syn1 punctae (r^2^ of 0.47 for mouse RNAi and 0.59 for negative control shRNA population), (B) between inhibitory post-synaptic Gphn and pre-synaptic Syn1 punctae (r^2^ of 0.43 for mouse RNAi and 0.69 for negative control shRNA) and between (C) excitatory (Psd95-Syn1) and inhibitory synapse (Gphn-Syn1) density development (r^2^ of 0.73 for mouse genes and 0.76 for negative control). shPsd95 specifically reduces excitatory synapse density, shGphn reduces inhibitory synapse density and shSyn affects both. The 2 novel regulators of synapse development identified in this screen are Axin1 and Grin2C (see also Fig. 4). (D) Regression analysis of excitatory-inhibitory synapse development presented in (C) highlights the specificity of shPsd95 and shGphn for excitatory and inhibitory synapse development. The quantitative results and statistical analysis of all 5 shRNAs targeting each gene is presented in Fig. S5, S10 and S11 and Table S2 and S3.

The automated image analysis platform makes possible analyses of individual shRNAs and the population of RNAis in the library to discern effects on specific neuronal phenotypes across 33,400 acquired images. For instance, we observed enrichment of mouse genes reducing dendrite development compared to the control negative hairpin population (Fig. S7; p = 0.0348, 2-tailed t-test). Importantly, the absence of correlation between synapse development and dendrite outgrowth (Fig. S8) indicates that differences in synapse density were not caused by changes in gross development. The similar distributions of shRNAs targeting mouse genes and the negative controls (Fig. S9) suggest that the majority of shRNAs do not regulate excitatory (p = 0.6944, 2-tailed *t*-test) or inhibitory synapse development (p = 0.2515, 2-tailed *t*-test). Furthermore, linear regression analysis indicates a high degree of correlation between the development of post-synaptic excitatory Psd95 and pre-synaptic Syn1 punctae (Fig 3A), between post-synaptic inhibitory Gphn and pre-synaptic Syn1 punctae (Fig 3B) and between excitatory and inhibitory synapses (Fig. 3C, D). Noted exceptions are positive control hairpins with moderate knockdown effect on Psd95 and Gphn expression that specifically affected excitatory or inhibitory neurons respectively (Fig. 2, 3, Table S2 and S3). This is further highlighted by cumulative frequency distributions of the effect of shPsd95, shGphn, shSyn1 and shTsc1 on excitatory and inhibitory synapse development relative to negative controls and the mouse library (Fig. S9). This demonstrates that the image analysis algorithm permits unbiased analysis of a large number of perturbations on several complex phenotypic aspects of synaptogenesis.

### Identification of novel regulators of synapse development

**Figure 4:**
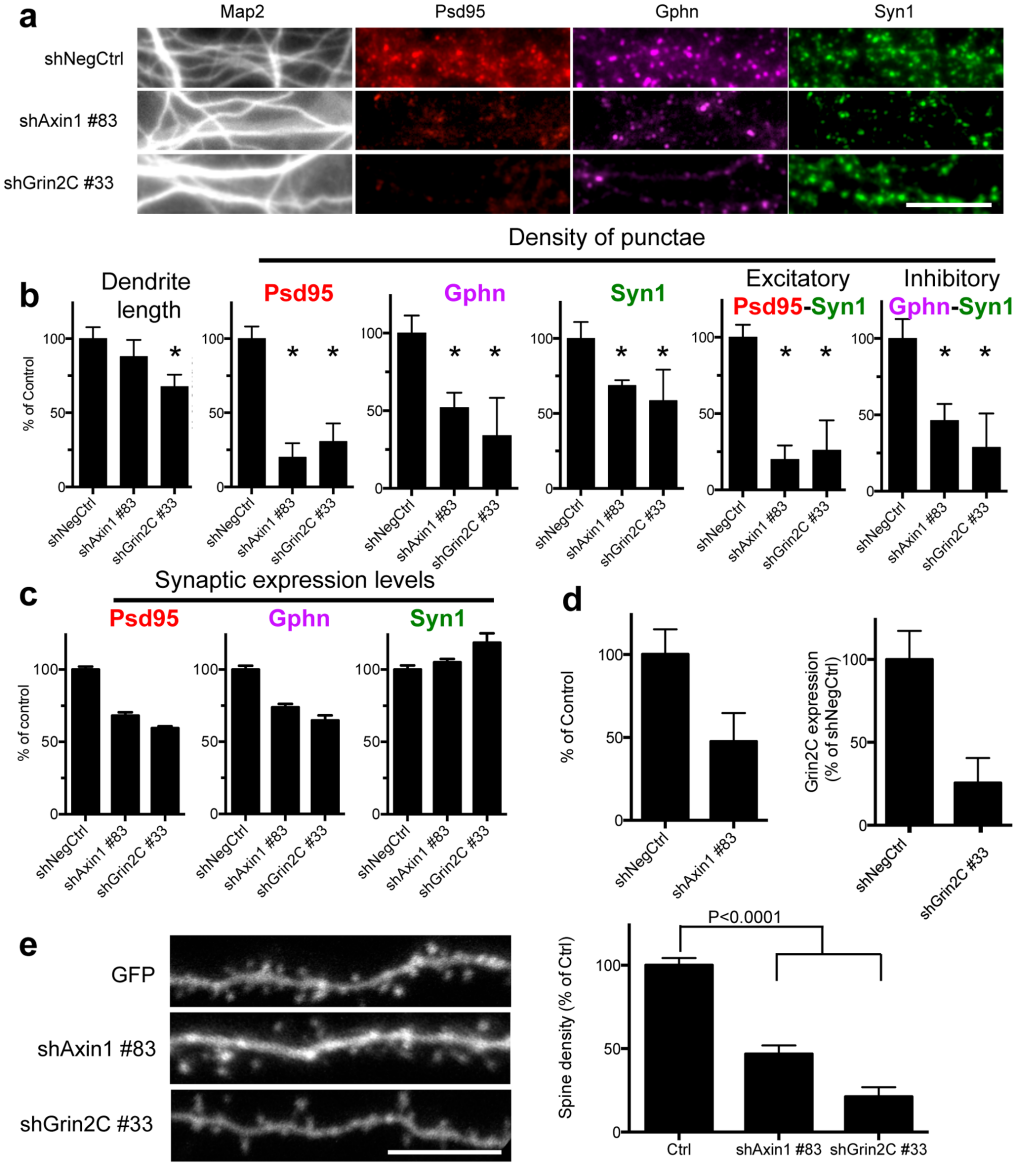
Identification of novel regulators of synapse development in the RNAi screen. (A) Representative selected areas of immunofluorescence images taken with a 40X objective of dendrites and synapses of negative control (shNegCtrl; shLacZ#30) and the most effective shRNAs targeting Axin1 and Grin2C, and the quantification thereof (B) (median and median absolute deviations of primary screen and two independent secondary tests). Asterisks denote p<0.05 or less (2-tailed t-test). The scale bar represents 10 μm. (C) shRNAs also reduce synaptic expression levels of Psd95 and Gphn. (D) Confirmation of the on-target knockdown of shAxin1 and shGrin2C by western blot. (E) Orthologous spine density assays experiments visually and quantitatively confirm that shRNA to Axin1 and Grin2C reduced spine density. p<0.0001 (2-tailed T-test). Fig. S10 and S11 and Tables S2 and S3 present data of all 5 shRNAs targeting these genes.

We applied the image analysis algorithm to identify novel regulators of synapse development in the RNAi screen that targeted 118 genes. The path of RNAi-to-gene validation we describe aimed to maximize discovery of on target hits by prioritizing potential hit genes by (1) the size-effect on synapse density (reduction of synaptogenesis by ≧40%), (2) the number of shRNA sequences that produce the same phenotype (2 of 5 shRNA reducing synaptogenesis by ≧40%) (3) reproducibility (shRNA effect repeated in 3 independent screens with minimal variability) and (4) visual confirmation of the phenotype of the candidate genes accordingly highlighted by the imaging algorithm. This nominated Axin1 and Grin2C as potential novel regulators of synaptogenesis, visually (Fig 4A and Fig. S2, S3, S4) and quantitatively (Fig 3, 4B,C and Fig S10 A–C and S11 A–C). This does not exclude the possibility that single shRNA hits (e.g. Axin2 (Fig. S12) or multiple hairpin hits with weak effects may also nominate potential regulators of synapse development (e.g. Tsc1, Fig. 2 and Fig. S5).

ShRNA-mediated suppression of Grin2C equally impinges on the formation of all classes of synaptic boutons (one way ANOVA, Table S3). However, Axin1 preferentially regulates the development of excitatory post-synaptic Psd95 and Psd95-Syn1 punctae, with only modest or little effect on Gphn and Syn1 punctae development (one way ANOVA, Table S3).

Importantly, the on-target specificity of RNAi reagents was confirmed by shRNA mediated protein knockdown of Axin1 and Grin2C, which correlated with the defective synapse phenotype (Fig. 4D, Fig. S10D, S11D). Finally, the synapse reducing effect of positive control shPsd95 and shTsc1 (Fig. S13) and shAxin1 and shGrin2C (Fig. 4E) was confirmed in orthologous spine assays in acute brain slices. The discovery of novel candidate positive regulators of synaptogenesis with general – Grin2C- or specific effects on excitatory synaptogenesis – Axin1 - is a further demonstration of the utility of the functional genomic screening methodology and the synapse imaging algorithm.

## Discussion

The high-throughput screening methodology presented here enables robust and reproducible unbiased interrogation of the molecular pathways underlying synapse development in primary culture of mouse neurons. This provides a valuable system for the study of normal developmental and activity dependent synapse formation as well as perturbations resulting from disease-associated gene mutations. We present protocols to achieve neuronal lentiviral infection rates of near 100% and little variation among replicate RNAi perturbations, an indication that scale up for genome-wide functional screens and large-scale drug screens of synaptogenesis is feasible.

Previous successful efforts towards screening of genes that regulate synapse formation have relied on heterologous systems[11], low-efficiency lipid-based transfections of siRNAs that require visual identification of the affected neurons and manual analysis of synapse number[12], or indirect measures of synapse formation such as fluorescence intensity rather than identification of individual synaptic punctae[13]. By contrast, to our knowledge, this is the first example of a quantitative automated screen for endogenous synapse development in primary neuronal cultures using unbiased and unsupervised image analysis.

Rich information can be harvested from this assay using the quantitative automated image analysis algorithm presented here, which is freely available and built on open-source software. The discovery of genes regulating the development of specific (e.g. pre v.s post) synaptic compartments is one important example of the power of the RNAi and image analysis methodology presented. This tool can parse several aspects of synapse development, including synapse size, synaptic protein expression levels and composition of synapses, as well as dendrite outgrowth. In the RNAi screen we identify 2 novel regulators of synapse development, Axin1 and Grin2C. These genes are components of pathways that are known modulators of the biology of synapses: Wnt pathway for Axin1 and NMDA receptors for Grin2C[14]–[16]. The lack of direct interaction that has previously been observed between Grin2C and PSD95 PDZ domains[17] may be offered as one explanation for defects in synaptogenesis. In addition, since Grin2C is expressed early postnatally and NMDA receptors containing this subunit display less voltage-dependent block by extracellular magnesium, Grin2C may play a privileged role in early synaptogenesis[18], [19]. Ostensibly, the scaffold protein Axin is localized at PSD95 enriched post-synaptic density fractions[15] and may regulate several key protein complexes that govern synaptogenesis, spine morphogenesis and synaptic function. Examples of these complexes include Gsk3B/APC/B-catenin, B-catenin/Cadherin, S-SCAM/Stargazin/AMPA-R/Gsk3B/Bcatenin, Grb4/Ephrin-B/GIT1/Actin, and Cdk5[14], [20]–[23]. Competing for Axin1 availability under conditions of partial loss of expression, dysregulation of all these complexes may underlie impaired synapse formation. Additional experiments are needed to begin to elucidate the mechanisms by which Grin2C and Axin1 may regulate synaptogenesis.

A crucial feature of the image analysis algorithm is the ability to separately analyze punctae of different classes, which can be used to study the intramolecular composition of synaptic compartments. These punctae can be visualized with multiplexed antibodies (or proteins fused with fluorescent proteins) labeled with fluorophores with different spectra. Here we used the algorithm for functional genomic analysis of the development of pre- and post-synaptic terminals and of excitatory and inhibitory synapses. A direct disease relevance to the study of these particular classes of synapses is the emerging view that certain autism spectrum disorders (ASDs) and schizophrenia may be caused by an imbalance in the development and/or activity of excitatory and inhibitory synapses[24]. Moreover, de novo CNV analyses indicate an enrichment of genes of the PSD95 complex in schizophrenia[25], and rare exonic deletions implicate Gephyrin as a risk factor for autism, schizophrenia and seizures[26]. The recent discovery of de novo CNV deletions of Axin1[27] and exome variants of the Grin2C family member Grin2B associated with ASDs[27] further exemplify the utility of the synapse analysis pipeline in the functional analysis of risk genes of neurological disorders. Indeed, the RNAi screening and synapse quantitation methodology will be invaluable tools for the translation of genetic associations of human neurological disorders identified in genome wide association and exome sequencing studies (e.g. candidate risk genes of Alzheimer's disease, schizophrenia and autism spectrum disorders[28], [29]) into pathophysiological mechanisms.

Our RNAi screening protocols and secondary assay validation strategy should be amenable to the analysis of other neural cell types and phenotypes including developmental assays (e.g. axo-dendritic development), electrophysiological read-outs (e.g. 96-well microelectrode array set-up) and synaptic activity[30]. The imaging algorithm may be used for both *in-vitro* and *in-vivo* synaptic punctae analysis. Potential applications of our system include screening for drugs and genes that regulate or rescue synaptic disorders underlying Alzheimer's disease, autism spectrum disorders (e.g. syndromes of Fragile X, Tuberosclerosis, Rett and Angelman) and neuropsychiatric disorders (e.g. monogenetic DISC1 disorders, 22q11 syndrome).

## Supporting Information

Figure S1 **Overview of the high-throughput screening methodology of synaptogenesis.** This includes seeding and lentiviral infection of mouse primary neurons in 96-well format, selection of infected cells with puromycin, immunofluorescence staining and automated high content image analysis of synapses and dendrites. Quality control measurements confirmed infection efficiency, the effect of positive control shRNA on synapse development and absence of toxic effects. Genes were pursued in secondary experiments if 2 or more shRNAs in the primary screen reduced synapse development by 60% or more. Secondary experiments included retesting twice all 5 shRNAs targeting each gene in the identical synapse development screen, spine development assays in slice cultures and on-target knockdown confirmation by western blots.
(TIF)

Figure S2 **Pipeline of the synapse development algorithm.** Critical steps of the algorithm are displayed using a fluorescence image from a negative control shRNA LacZ#30 as an example. (A) Whole field fluorescence images of dendrite (A1–F1), Psd95 (A2–E2), Gphn (A3–E3) and Syn1 (A4–E4) were acquired with a high-throughput microscope using a 40X objective. Scale bar denotes 25 μm. Successive image processing steps include a background illumination correction (B1–B4), the results of which are showed in C1–C4, followed by image thresholding (D1–D4), object segmentation (E1–E4), skeletonization of the dendrites (F) and identification of overlapping Psd95-Syn1 and Gphn-Syn1 synaptic punctae pairs shown superimposed on the dendrite mask (G). This was instrumental in calculating synapse area, synaptic protein expression and density of synapse (pairs) and the dendrite length per image. Data is aggregated across all images of each individual well and then of replicate wells of the same shRNA sequences to obtain the median, median absolute deviation and % coefficient of variation.
(TIF)

Figure S3 **Microscopy analysis of shRNA induced changes in synapse development.** Whole field images taken with a 40X objective of representative wells infected with lentiviruses encoding negative, positive control shRNA and two novel synapse regulators. Relative to the negative control hairpin LacZ#30 (A1–A4), unique individual hairpin sequences targeting Psd95 (B1–B4, C1–C4), Gphn (D1–D4), Syn1 (E1–E4) show the expected decrease in development of Psd95, Gphn and Syn1 punctae respectively. RNAi of Tsc1 (F1–F4) reduced post-synaptic Psd95 and Gphn punctae and pre-synaptic Syn1 punctae. (G1–G4) and (H1–H4) display the effect of shRNA targeting the novel synapse regulators Axin1 (G) and Grin2C (H). Scale bar denotes 25 μm. Fig. 2 and 4 presents magnifications of representative selected areas of each of these images.
(TIF)

Figure S4 **Segmentation masks of RNAi induced changes in synapse development identified by the synapse imaging algorithm.** Segmentation masks of images presented in Fig. S3 demonstrate that the algorithm is able to successfully identify changes in Psd95 (A2–H2), Gphn (A3–H3), Syn1 (A4–H4) and overlapping Psd95-Syn1 and Gphn-Syn1 punctae (A5–H5) induced by shPsd95 (B1–B5), shGphn (C1–C5), shSyn1 (D1–D5), shTsc1 (E1–E5), shAxin1 (F1–F5) and shGrin2C (G1–G5) in comparison to the negative control (A1–A5). Scale bar denotes 25 μm.
(TIF)

Figure S5 **Quantification of RNAi induced changes in synapse development measured by automated image analysis.** The effects (median and median absolute deviations) on dendrite outgrowth (A), synapse development (B–F) and synaptic protein expression (mean and SD of primary screen results only) (G–I) of all 5 shRNA targeting Psd95, Gphn, Synapsin1 and Tsc1 was quantified using the synapse development algorithm. Data presented is the average of the primary screen and 2 independent secondary experiments. *p-values <0.05 or less (2-tailed t-tests, see table S2). This figure highlights the specificity of shPsd95, shGphn and shSyn1 on the development of (B) Psd95, (C) Gphn and (D) Syn1 punctae respectively.
(TIF)

Figure S6 **Frequency distribution of % coefficient of variation of the effect of shRNAs on synapse and dendrite development in the primary screen.** The averages and %CV was calculated in the primary screen for replicates of each of the 597 mouse and 80 negative control shRNAs (3–5 replicates per shRNA) in the library. Frequency distribution histograms of %CV of (A) number and (B) density of synaptic punctae, (C) number and (D) density of excitatory Psd95-Syn1 and Gphn-Syn1 punctea and (E) dendrite length demonstrate the robustness of the culture, infection and image analysis methodology (median %CV of 6.8% or less for these features).
(TIF)

Figure S7 **Cumulative frequency distribution of the effect of shRNAs on dendritic development in the primary synapse screen.** The dendrite outgrowth module of our synapse analysis algorithm identified an enrichment of hairpins reducing dendrite development in the library of 623 shRNAs targeting mouse genes (MsGenes) compared to the 80 negative control non-targeting shRNAs (p = 0.0348, 2 tailed t-test).
(TIF)

Figure S8 **Absence of correlation between dendrite development and density of individual synaptic punctae.** Primary screen analysis of the effect of 597 mouse shRNAs (shMsGenes) and 20 positive control shRNAs and 80 negative control hairpins (shNegCtrl) show little correlation between the effect of shRNAs on dendrite development and the density of (A) Psd95 (R^2^ of 0.3296 and 0.4226 for shMsGenes and shNegCtrl, respectively), (B) Syn1 (R^2^ of 0.1038 and 0.2327 for shMsGenes and shNegCtrl, respectively) and (C) Gphn (R^2^ of 0.2361 and 0.3971 for shMsGenes and shNegCtrl, respectively) punctae. The symbols present the median results of replicate shRNA infection. The median absolute deviation was plotted for the positive control shRNAs to Psd95, Syn1, Gphn and Tsc1.
(TIF)

Figure S9 **Cumulative frequency distribution of the effect of shRNAs on the development of excitatory and inhibitory synapses.** The cumulative frequency distribution of the primary screen highlights the specificity of shPsd95, shGphn, shSyn1 and shTsc1 on the development of Psd95-Syn1 excitatory (A) and Gphn-Syn1 inhibitory punctae. (B) Shown are averaged effects of 5 positive control shRNA targeting Psd95, Gphn, Syn1 and Tsc1 in comparison with 80 individual negative control shRNA and 597 shRNAs targeting the mouse gene library.
(TIF)

Figure S10 **Identification by image analysis and western blot of Axin1 as regulator of synapse development.** (A) The effect of all 5 shRNA reagents targeting Axin1 on synapse development and dendrite outgrowth was quantified using the synapse imaging algorithm (*p-values <0.05 or less (2-tailed t-tests, see table S2)) (B) shRNAs also reduce synaptic protein expression (mean and SD of primary screen results only) and (C) development of excitatory and inhibitory synapses. (D) shRNAs that reduced synapse development also reduced expression of Axin1 in DIV14 cortical mouse neuronal cultures confirming the on-target specificity of these RNAi. (A) and (D) present median and median absolute deviations of the primary screen and two secondary screens. Statistical analysis is presented in Table S2 and S3.
(TIF)

Figure S11 **Identification by image analysis and western blot of Grin2C as regulator of synapse development.** (A) The effect of all 5 shRNA reagents targeting Grin2C on synapse development and dendrite outgrowth was quantified using the synapse imaging algorithm (*p-values <0.05 or less (2-tailed t-tests, see table S2)) (B) shRNAs also reduce synaptic protein expression (mean and SD of primary screen results only) and (C) development of excitatory and inhibitory synapses. (D) shRNAs that reduced synapse development also reduced expression of Axin1 in DIV14 cortical mouse neuronal cultures confirming the on-target specificity of these RNAi. (A) and (D) present median and median absolute deviations of the primary screen and two secondary screens. Statistical analysis is presented in Table S2 and S3.
(TIF)

Figure S12 **Effect on synapse development of shRNAs targeting Axin2.** (A) The effect of all 5 shRNA reagents targeting Axin2 on synapse development and dendrite outgrowth was quantified using the synapse imaging algorithm (*p-values <0.05 or less (2-tailed t-tests, see table S2)) (B) shRNAs also reduce synaptic protein expression (mean and SD of primary screen results only) and (C) development of excitatory and inhibitory synapses. Data present the average data of the primary RNAi screen. Statistical analysis is presented in Table S2 and S3.
(TIF)

Figure S13 **Regulation of spine density by shRNAs targeting Psd95 and Tsc1.** Orthologous spine density assays experiments confirm that shRNA to Psd95 #80 and Tsc1 #49 reduce spine density qualitatively (A) and quantitatively (B) p<0.0001 (2-tailed T-test compared to GFP expressing vector) Scale bar denotes 10 μm.
(TIF)

Table S1 **Content of the shRNA lentiviral library.** The library consists of 607 shRNA targeting mouse genes (shMsGenes), a negative control library of 80 unique non-targeting shRNA (shGFP, shRFP, shLuc, shLac) and a set of 10 negative control shRNAs infected as a pool in secondary synapse screens and for western blot experiments.
(XLSX)

Table S2 **T-test (2-tailed) of the effect of positive control shRNA on synapse development in comparison to negative control hairpins.** This demonstrates statistically significant changes (p-values in density of Psd95, Gphn, Syn1, Psd95-Syn1 and Gphn-Syn1 punctae, and dendrite length, by positive control shRNAs (shPSD95, shGphn, shSyn1, shTsc1) and shRNAs targeting Axin1, Grin2C and Axin2.
(XLSX)

Table S3 **Analysis, by one way ANOVA with Bonferroni's multiple comparisons correction, of the effects of positive control shRNAs on synapse development of the opposing class.** This examines statistical significance of the specificity of RNAi mediated knockdown on the density of the intended target (in comparison with the other 2 synaptic punctae). That is, shPsd95 preferentially suppresses Psd95 punctae, shGphn targets Gphn punctae and shSyn1 targets Syn1 punctae. No statistically significant difference was observed for the effect of shTsc1 or shGrin2C on Psd95, Gphn and Syn1 density. In contrast, 2 shRNAs targeting Axin1 significantly suppress the density of post synaptic-excitatory Psd95 punctae density compared to post synaptic-inhibitory Gphn punctae and pre-synaptic Syn1 punctae.
(XLSX)

## References

[1] Waites CL, Craig AM, Garner CC (2005) Mechanisms of vertebrate synaptogenesis. Annual Review of Neuroscience 28: 251–274. 10.1146/annurev.neuro.27.070203.144336 16022596

[2] Boda B, Dubos A, Muller D (2010) Signaling mechanisms regulating synapse formation and function in mental retardation. Current Opinion in Neurobiology 20: 519–527. 20413294 10.1016/j.conb.2010.03.012

[3] Penzes P, Cahill ME, Jones KA, VanLeeuwen JE, Woolfrey KM (2011) Dendritic spine pathology in neuropsychiatric disorders. Nature Neuroscience 14: 285–293. 21346746 10.1038/nn.2741 PMC3530413

[4] Ting JT, Peca J, Feng GP (2012) Functional Consequences of Mutations in Postsynaptic Scaffolding Proteins and Relevance to Psychiatric Disorders. Annual Review of Neuroscience, Vol 35 35: 49–71. 10.1146/annurev-neuro-062111-150442 22540979

[5] Root DE, Hacohen N, Hahn WC, Lander ES, Sabatini DM (2006) Genome-scale loss-of-function screening with a lentiviral RNAi library. Nature Methods 3: 715–719. 16929317 10.1038/nmeth924

[6] Carpenter AE, Jones TR, Lamprecht MR, Clarke C, Kang IH, et al. (2006) CellProfiler: image analysis software for identifying and quantifying cell phenotypes. Genome Biology 7.. 10.1186/gb-2006-7-10-r100 PMC1794559 17076895

[7] Schneider CA, Rasband WS, Eliceiri KW (2012) NIH Image to ImageJ: 25 years of image analysis. Nature Methods 9: 671–675. 22930834 10.1038/nmeth.2089 PMC5554542

[8] Sato YNS, Shiraga N, Atsumi H, Yoshida S, Koller T, et al (1998) Three-dimensional multi-scale line filter for segmentation and visualization of curvilinear structures in medical images. Medical image analysis 2: 143–168. 10646760 10.1016/s1361-8415(98)80009-1

[9] Otsu N (1979) A threshold selection method from gray-level histograms. IEEE Transactions on Systems, Man and Cybernetic 9: 62–66.

[10] Alvarez VA, Sabatini BL (2007) Anatomical and physiological plasticity of dendritic spines. Annual Review of Neuroscience 30: 79–97. 10.1146/annurev.neuro.30.051606.094222 17280523

[11] Linhoff MW, Lauren J, Cassidy RM, Dobie FA, Takahashi H, et al (2009) An Unbiased Expression Screen for Synaptogenic Proteins Identifies the LRRTM Protein Family as Synaptic Organizers. Neuron 61: 734–749. 19285470 10.1016/j.neuron.2009.01.017 PMC2746109

[12] Paradis S, Harrar DB, Lin YX, Koon AC, Hauser JL, et al (2007) An RNAi-based approach identifies molecules required for glutamatergic and GABAergic synapse development. Neuron 53: 217–232. 17224404 10.1016/j.neuron.2006.12.012 PMC1950560

[13] Shi P, Scott MA, Ghosh B, Wan DP, Wissner-Gross Z, et al. (2011) Synapse microarray identification of small molecules that enhance synaptogenesis. Nature Communications 2.. 10.1038/ncomms1518 PMC3544154 22027590

[14] Brigidi GS, Bamji SX (2011) Cadherin-catenin adhesion complexes at the synapse. Current Opinion in Neurobiology 21: 208–214. 21255999 10.1016/j.conb.2010.12.004

[15] Chen Y, Fu AK, Ip NY (2013) Axin: An emerging key scaffold at the synapse. Iubmb Life 65: 685–691. 23847014 10.1002/iub.1184

[16] Sanhueza M, Lisman J (2013) The CaMKII/NMDAR complex as a molecular memory. Molecular Brain 6: 1–8. 23410178 10.1186/1756-6606-6-10 PMC3582596

[17] Kornau HC, Schenker LT, Kennedy MB, Seeburg PH (1995) Domain interaction between NMDA receptor subunits and the postsynaptic density protein PSD-95. Science 269: 1737–1740. 7569905 10.1126/science.7569905

[18] Monyer H, Burnashev N, Laurie DJ, Sakmann B, Seeburg PH (1994) Developmental and regional expression in the rat brain and functional properties of four NMDA receptors. Neuron 12: 529–540. 7512349 10.1016/0896-6273(94)90210-0

[19] Wenzel A, Fritschy JM, Mohler H, Benke D (1997) NMDA receptor heterogeneity during postnatal development of the rat brain: differential expression of the NR2A, NR2B, and NR2C subunit proteins. J Neurochem 68: 469–478. 9003031 10.1046/j.1471-4159.1997.68020469.x

[20] Bamji SX, Shimazu K, Kimes N, Huelsken J, Birchmeier W, et al (2003) Role of beta-catenin in synaptic vesicle localization and presynaptic assembly. Neuron 40: 719–731. 14622577 10.1016/s0896-6273(03)00718-9 PMC2757419

[21] Fang WQ, Ip JP, Li R, Ng YP, Lin SC, et al (2011) Cdk5-mediated phosphorylation of Axin directs axon formation during cerebral cortex development. Journal of Neuroscience 31: 13613–13624. 21940452 10.1523/JNEUROSCI.3120-11.2011 PMC6623306

[22] Samuels BA, Hsueh YP, Shu T, Liang H, Tseng HC, et al (2007) Cdk5 promotes synaptogenesis by regulating the subcellular distribution of the MAGUK family member CASK. Neuron 56: 823–837. 18054859 10.1016/j.neuron.2007.09.035 PMC2151975

[23] Okuda T, Yu LM, Cingolani LA, Kemler R, Goda Y (2007) beta-Catenin regulates excitatory postsynaptic strength at hippocampal synapses. Proc Natl Acad Sci U S A 104: 13479–13484. 17679699 10.1073/pnas.0702334104 PMC1948936

[24] Marin O (2012) Interneuron dysfunction in psychiatric disorders. Nat Rev Neurosci 13: 107–120. 22251963 10.1038/nrn3155

[25] Kirov G, Pocklington AJ, Holmans P, Ivanov D, Ikeda M, et al (2012) De novo CNV analysis implicates specific abnormalities of postsynaptic signalling complexes in the pathogenesis of schizophrenia. Molecular Psychiatry 17: 142–153. 22083728 10.1038/mp.2011.154 PMC3603134

[26] Lionel AC, Vaags AK, Sato D, Gazzellone MJ, Mitchell EB, et al (2013) Rare exonic deletions implicate the synaptic organizer Gephyrin (GPHN) in risk for autism, schizophrenia and seizures. Human Molecular Genetics 22: 2055–2066. 23393157 10.1093/hmg/ddt056

[27] Noh HJ, Ponting CP, Boulding HC, Meader S, Betancur C, et al (2013) Network topologies and convergent aetiologies arising from deletions and duplications observed in individuals with autism. Plos Genetics 9: e1003523. 23754953 10.1371/journal.pgen.1003523 PMC3675007

[28] Gejman PV, Sanders AR, Kendler KS (2011) Genetics of Schizophrenia: New Findings and Challenges. Annual Review of Genomics and Human Genetics, Vol 12 12: 121–144. 10.1146/annurev-genom-082410-101459 21639796

[29] Buxbaum JD, Daly MJ, Devlin B, Lehner T, Roeder K, et al (2012) The Autism Sequencing Consortium: Large-Scale, High-Throughput Sequencing in Autism Spectrum Disorders. Neuron 76: 1052–1056. 23259942 10.1016/j.neuron.2012.12.008 PMC3863639

[30] Hempel CM, Sivula M, Levenson JM, Rose DM, Li B, et al (2011) A System for Performing High Throughput Assays of Synaptic Function. Plos One 6: e25999. 21998743 10.1371/journal.pone.0025999 PMC3187845

