# Supplementary figures and images for: High Content Image Analysis Identifies Novel Regulators of Synaptogenesis in a High-Throughput RNAi Screen of Primary Neurons
Source: PLoS One. 2014 Mar 14;9(3):e91744. doi: 10.1371/journal.pone.0091744 (PMC3954765; doi:10.1371/journal.pone.0091744)

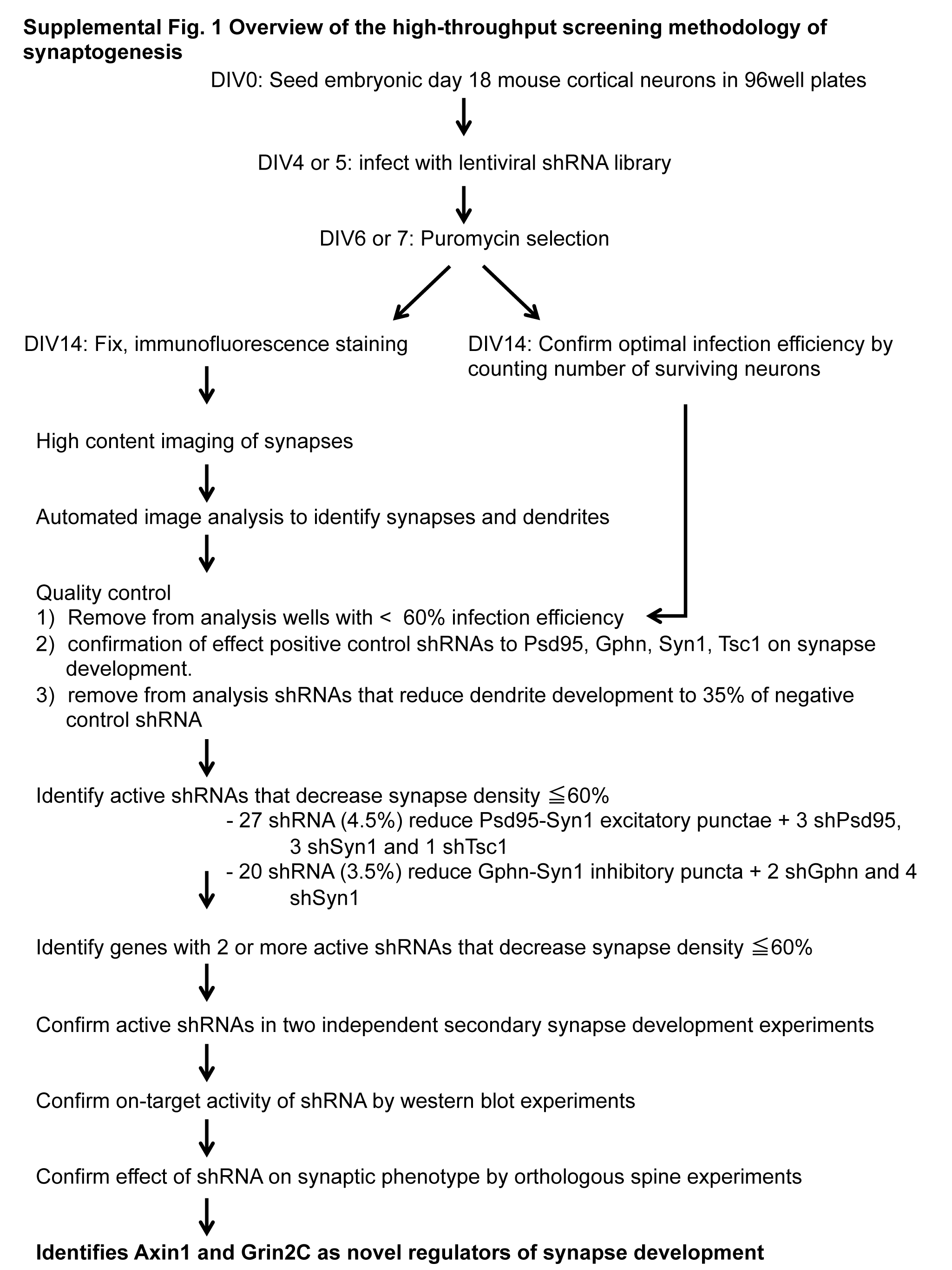

Supplement: Figure S1 — Overview of the high-throughput screening methodology of synaptogenesis. This includes seeding and lentiviral infection of mouse primary neurons in 96-well format, selection of infected cells with puromycin, immunofluorescence staining and automated high content image analysis of synapses and dendrites. Quality control measurements confirmed infection efficiency, the effect of positive control shRNA on synapse development and absence of toxic effects. Genes were pursued in secondary experiments if 2 or more shRNAs in the primary screen reduced synapse development by 60% or more. Secondary experiments included retesting twice all 5 shRNAs targeting each gene in the identical synapse development screen, spine development assays in slice cultures and on-target knockdown confirmation by western blots. (TIF) [file pone.0091744.s001.tif]

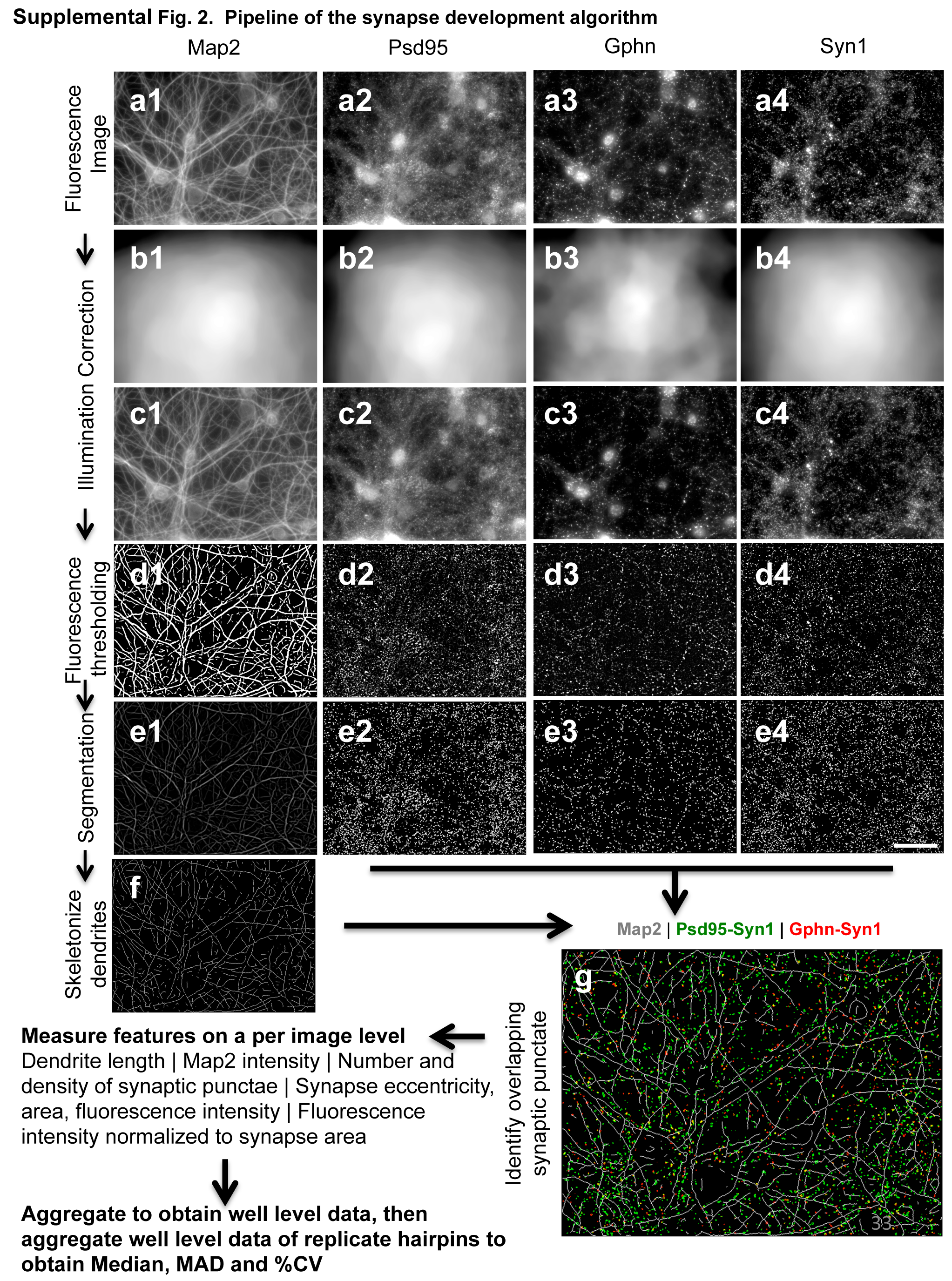

Supplement: Figure S2 — Pipeline of the synapse development algorithm. Critical steps of the algorithm are displayed using a fluorescence image from a negative control shRNA LacZ#30 as an example. (A) Whole field fluorescence images of dendrite (A1–F1), Psd95 (A2–E2), Gphn (A3–E3) and Syn1 (A4–E4) were acquired with a high-throughput microscope using a 40X objective. Scale bar denotes 25 μm. Successive image processing steps include a background illumination correction (B1–B4), the results of which are showed in C1–C4, followed by image thresholding (D1–D4), object segmentation (E1–E4), skeletonization of the dendrites (F) and identification of overlapping Psd95-Syn1 and Gphn-Syn1 synaptic punctae pairs shown superimposed on the dendrite mask (G). This was instrumental in calculating synapse area, synaptic protein expression and density of synapse (pairs) and the dendrite length per image. Data is aggregated across all images of each individual well and then of replicate wells of the same shRNA sequences to obtain the median, median absolute deviation and % coefficient of variation. (TIF) [file pone.0091744.s002.tif]

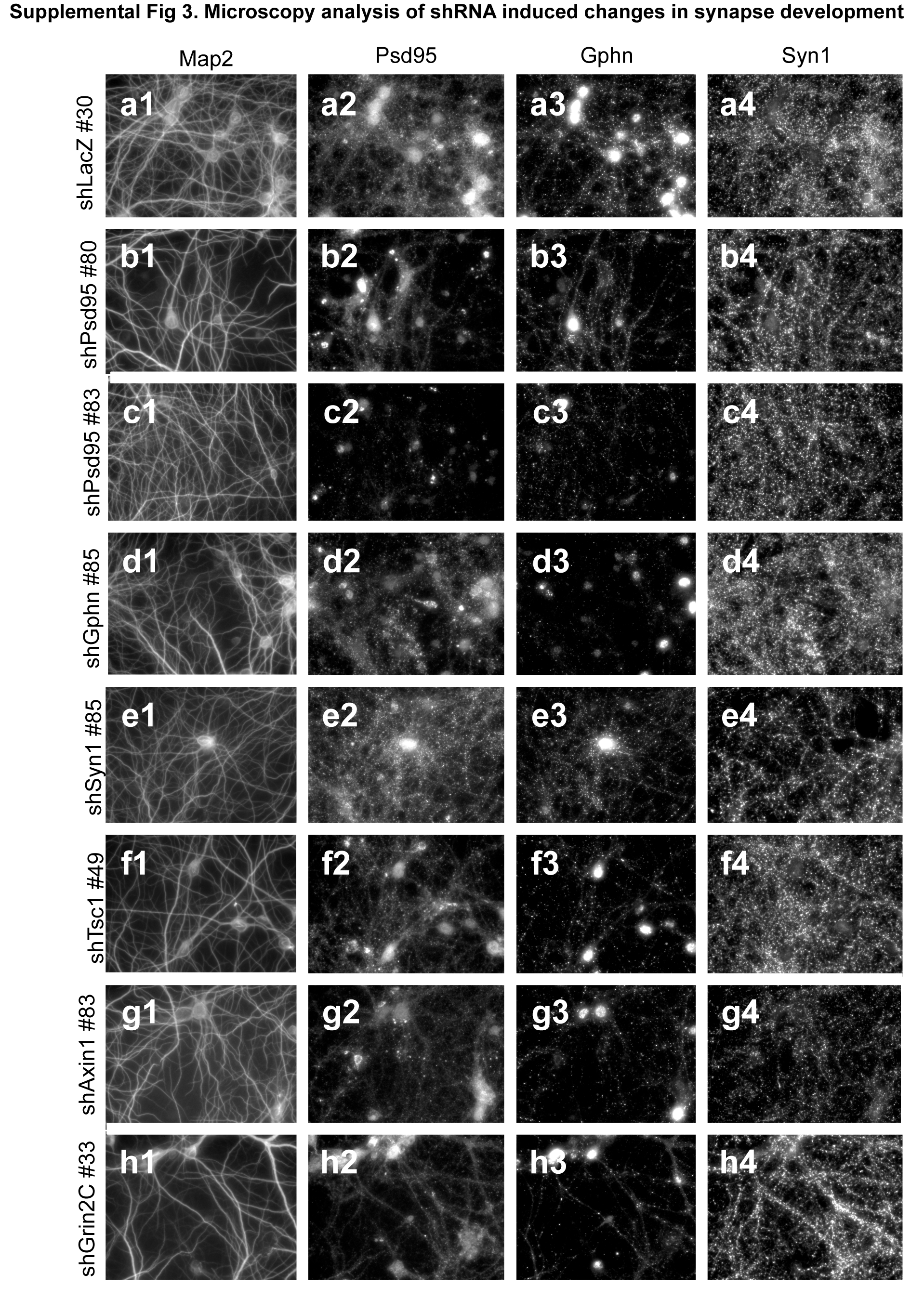

Supplement: Figure S3 — Microscopy analysis of shRNA induced changes in synapse development. Whole field images taken with a 40X objective of representative wells infected with lentiviruses encoding negative, positive control shRNA and two novel synapse regulators. Relative to the negative control hairpin LacZ#30 (A1–A4), unique individual hairpin sequences targeting Psd95 (B1–B4, C1–C4), Gphn (D1–D4), Syn1 (E1–E4) show the expected decrease in development of Psd95, Gphn and Syn1 punctae respectively. RNAi of Tsc1 (F1–F4) reduced post-synaptic Psd95 and Gphn punctae and pre-synaptic Syn1 punctae. (G1–G4) and (H1–H4) display the effect of shRNA targeting the novel synapse regulators Axin1 (G) and Grin2C (H). Scale bar denotes 25 μm. Fig. 2 and 4 presents magnifications of representative selected areas of each of these images. (TIF) [file pone.0091744.s003.tif]

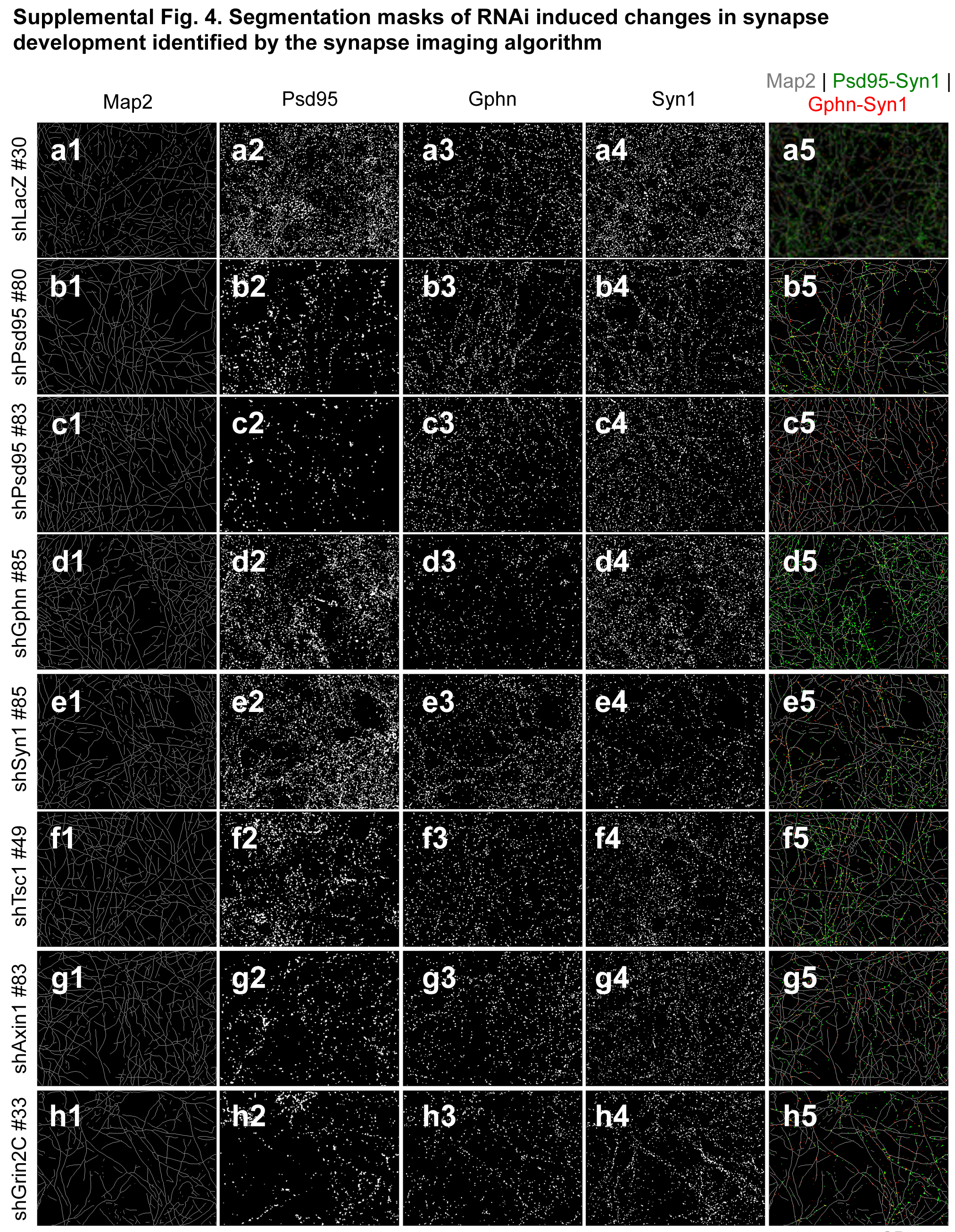

Supplement: Figure S4 — Segmentation masks of RNAi induced changes in synapse development identified by the synapse imaging algorithm. Segmentation masks of images presented in Fig. S3 demonstrate that the algorithm is able to successfully identify changes in Psd95 (A2–H2), Gphn (A3–H3), Syn1 (A4–H4) and overlapping Psd95-Syn1 and Gphn-Syn1 punctae (A5–H5) induced by shPsd95 (B1–B5), shGphn (C1–C5), shSyn1 (D1–D5), shTsc1 (E1–E5), shAxin1 (F1–F5) and shGrin2C (G1–G5) in comparison to the negative control (A1–A5). Scale bar denotes 25 μm. (TIF) [file pone.0091744.s004.tif]

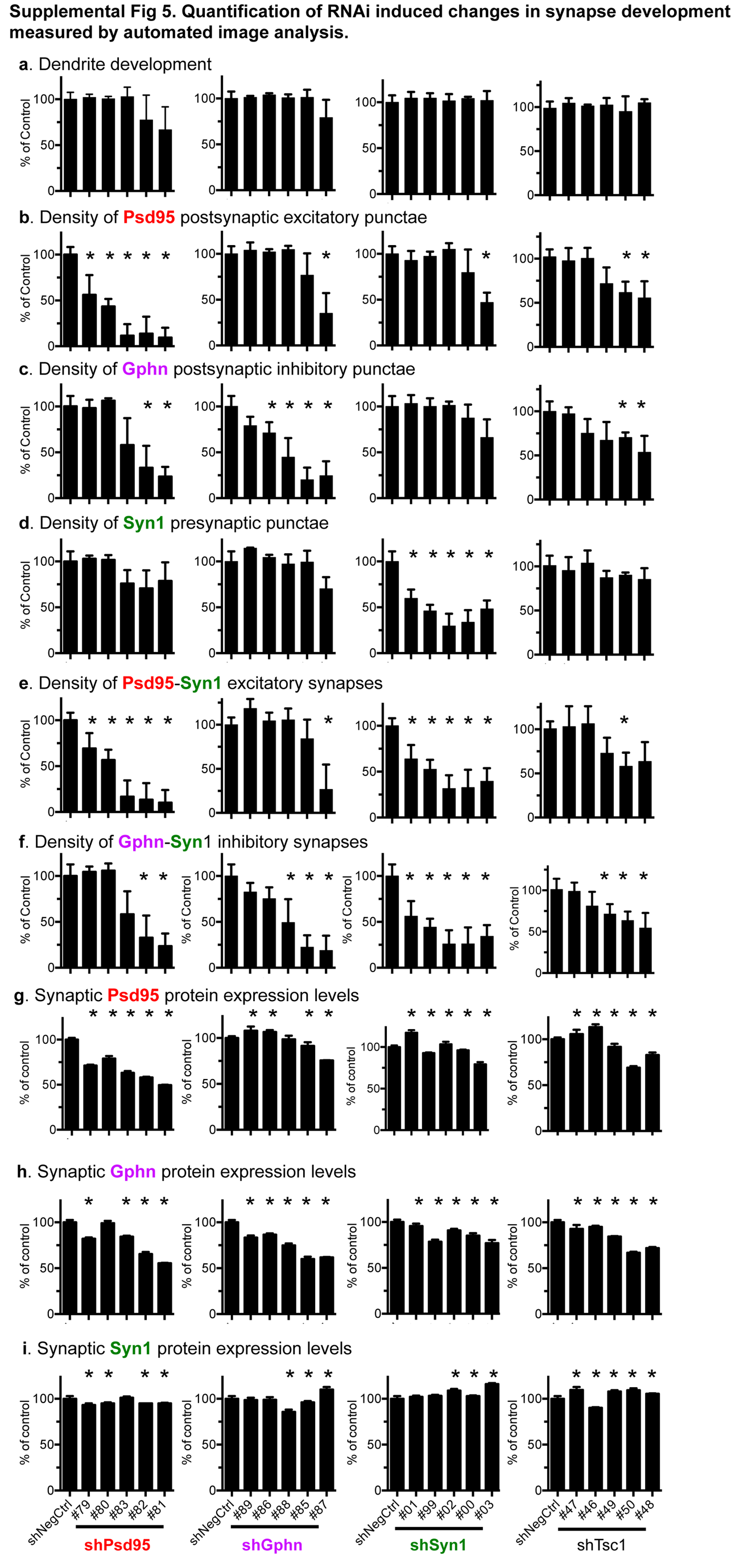

Supplement: Figure S5 — Quantification of RNAi induced changes in synapse development measured by automated image analysis. The effects (median and median absolute deviations) on dendrite outgrowth (A), synapse development (B–F) and synaptic protein expression (mean and SD of primary screen results only) (G–I) of all 5 shRNA targeting Psd95, Gphn, Synapsin1 and Tsc1 was quantified using the synapse development algorithm. Data presented is the average of the primary screen and 2 independent secondary experiments. *p-values <0.05 or less (2-tailed t-tests, see table S2). This figure highlights the specificity of shPsd95, shGphn and shSyn1 on the development of (B) Psd95, (C) Gphn and (D) Syn1 punctae respectively. (TIF) [file pone.0091744.s005.tif]

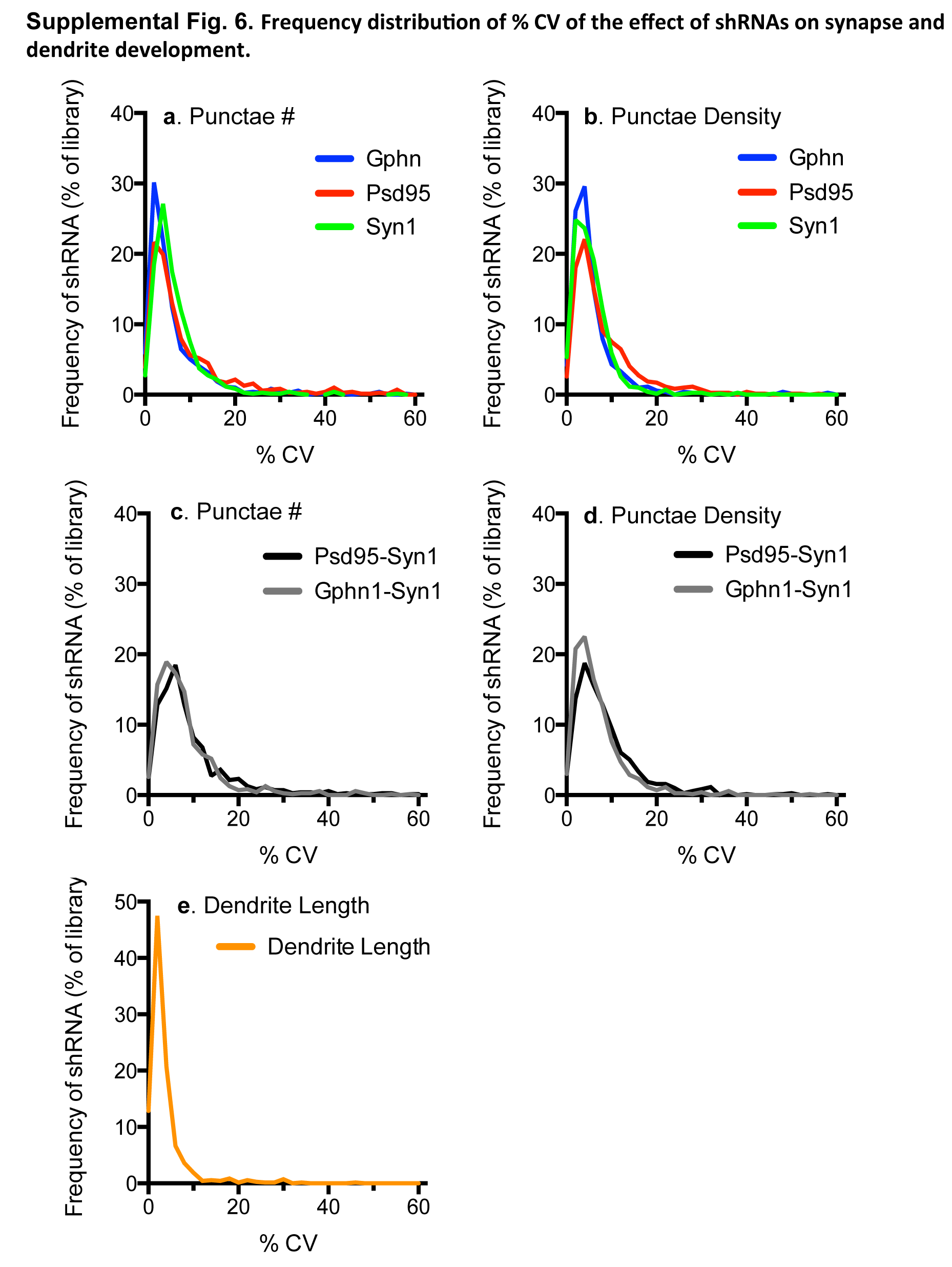

Supplement: Figure S6 — Frequency distribution of % coefficient of variation of the effect of shRNAs on synapse and dendrite development in the primary screen. The averages and %CV was calculated in the primary screen for replicates of each of the 597 mouse and 80 negative control shRNAs (3–5 replicates per shRNA) in the library. Frequency distribution histograms of %CV of (A) number and (B) density of synaptic punctae, (C) number and (D) density of excitatory Psd95-Syn1 and Gphn-Syn1 punctea and (E) dendrite length demonstrate the robustness of the culture, infection and image analysis methodology (median %CV of 6.8% or less for these features). (TIF) [file pone.0091744.s006.tif]

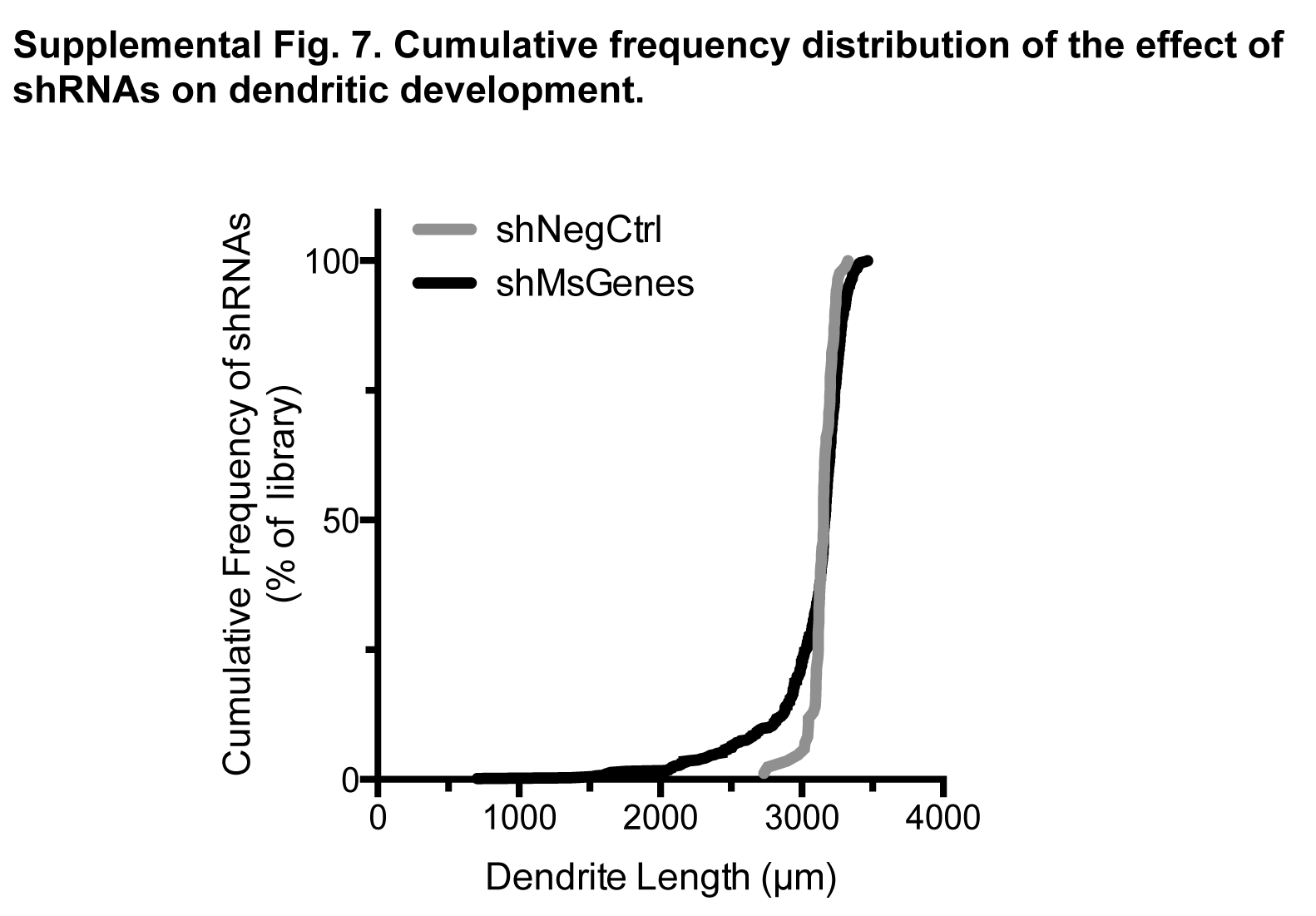

Supplement: Figure S7 — Cumulative frequency distribution of the effect of shRNAs on dendritic development in the primary synapse screen. The dendrite outgrowth module of our synapse analysis algorithm identified an enrichment of hairpins reducing dendrite development in the library of 623 shRNAs targeting mouse genes (MsGenes) compared to the 80 negative control non-targeting shRNAs (p = 0.0348, 2 tailed t-test). (TIF) [file pone.0091744.s007.tif]

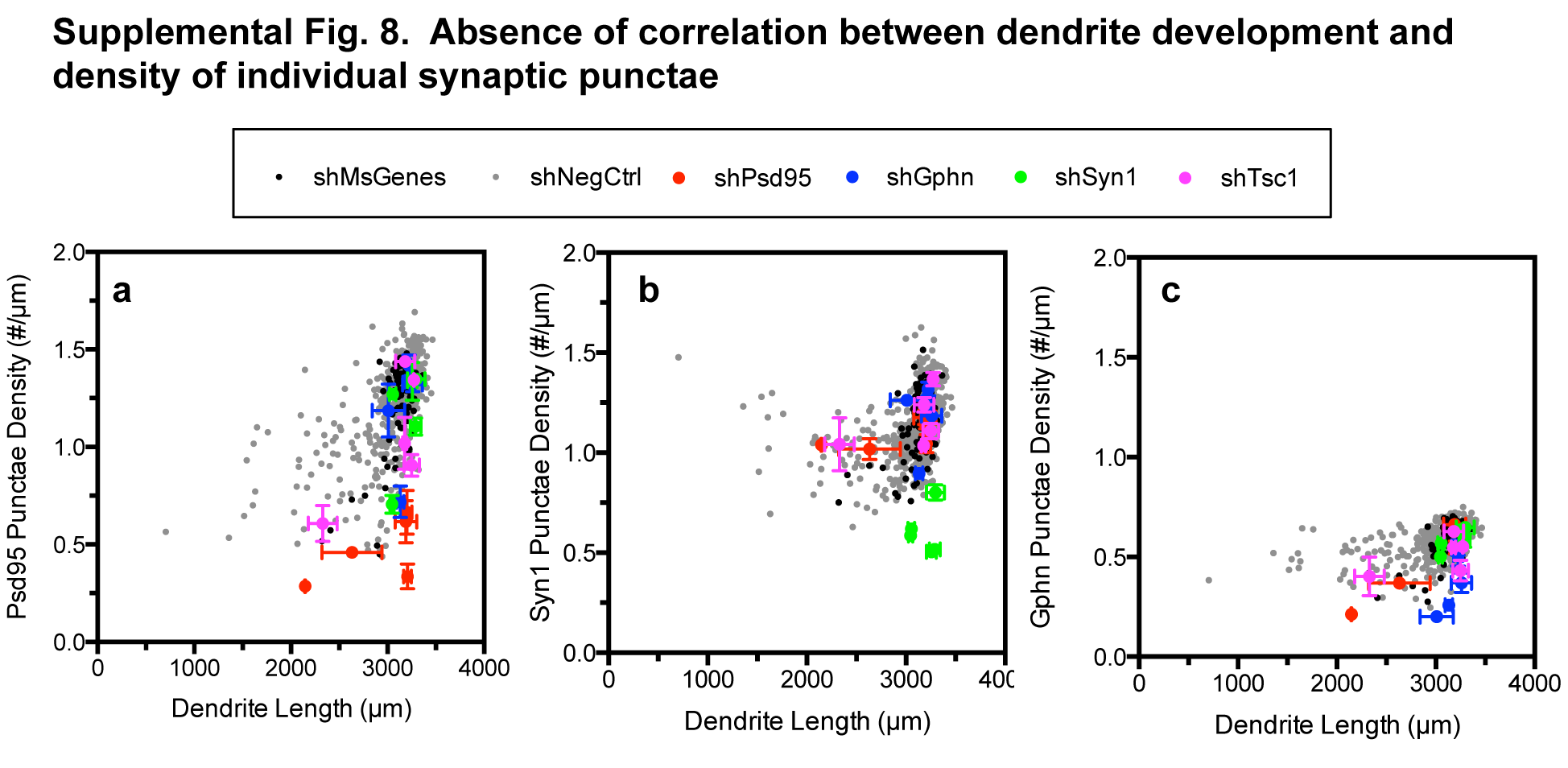

Supplement: Figure S8 — Absence of correlation between dendrite development and density of individual synaptic punctae. Primary screen analysis of the effect of 597 mouse shRNAs (shMsGenes) and 20 positive control shRNAs and 80 negative control hairpins (shNegCtrl) show little correlation between the effect of shRNAs on dendrite development and the density of (A) Psd95 (R2 of 0.3296 and 0.4226 for shMsGenes and shNegCtrl, respectively), (B) Syn1 (R2 of 0.1038 and 0.2327 for shMsGenes and shNegCtrl, respectively) and (C) Gphn (R2 of 0.2361 and 0.3971 for shMsGenes and shNegCtrl, respectively) punctae. The symbols present the median results of replicate shRNA infection. The median absolute deviation was plotted for the positive control shRNAs to Psd95, Syn1, Gphn and Tsc1. (TIF) [file pone.0091744.s008.tif]

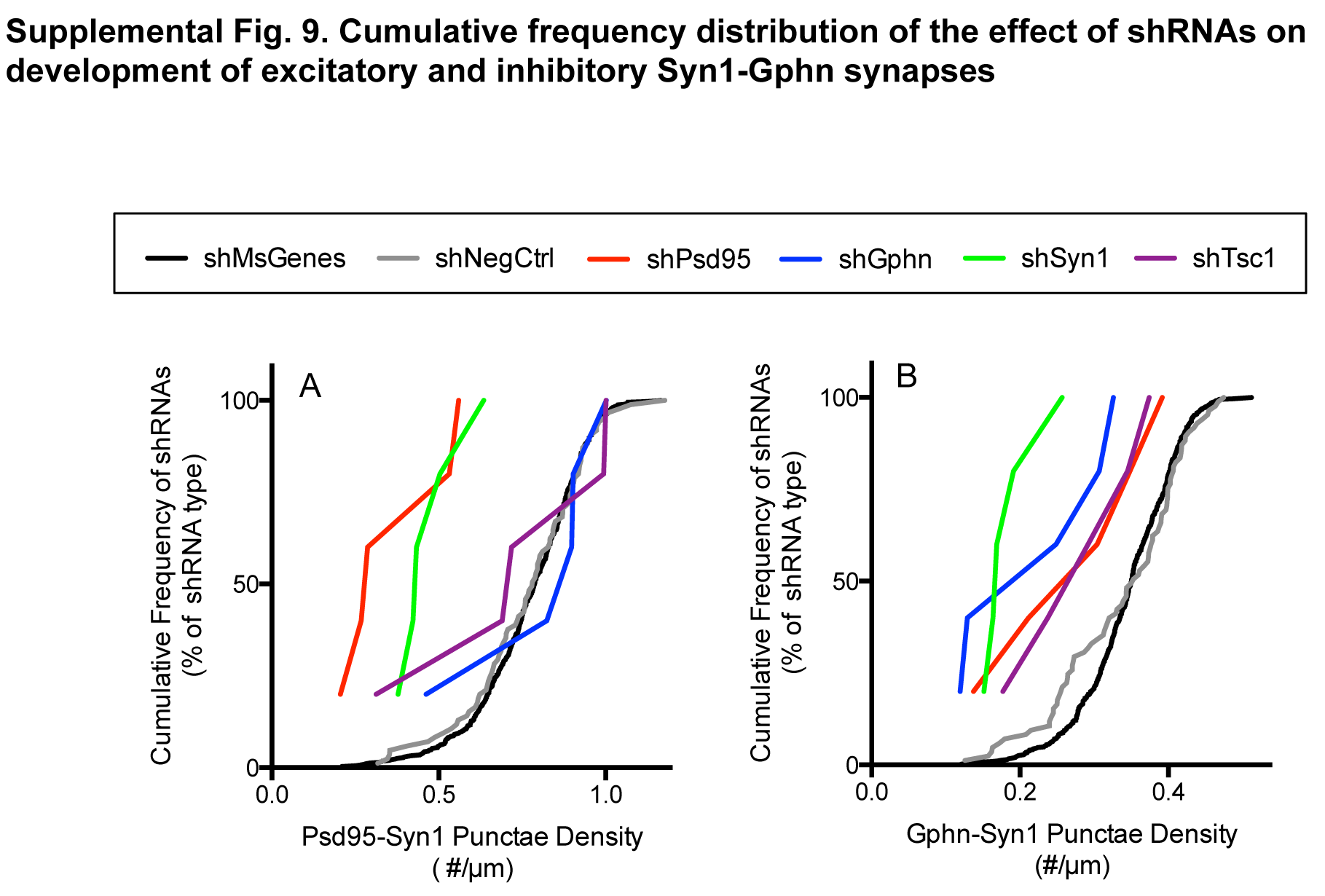

Supplement: Figure S9 — Cumulative frequency distribution of the effect of shRNAs on the development of excitatory and inhibitory synapses. The cumulative frequency distribution of the primary screen highlights the specificity of shPsd95, shGphn, shSyn1 and shTsc1 on the development of Psd95-Syn1 excitatory (A) and Gphn-Syn1 inhibitory punctae. (B) Shown are averaged effects of 5 positive control shRNA targeting Psd95, Gphn, Syn1 and Tsc1 in comparison with 80 individual negative control shRNA and 597 shRNAs targeting the mouse gene library. (TIF) [file pone.0091744.s009.tif]

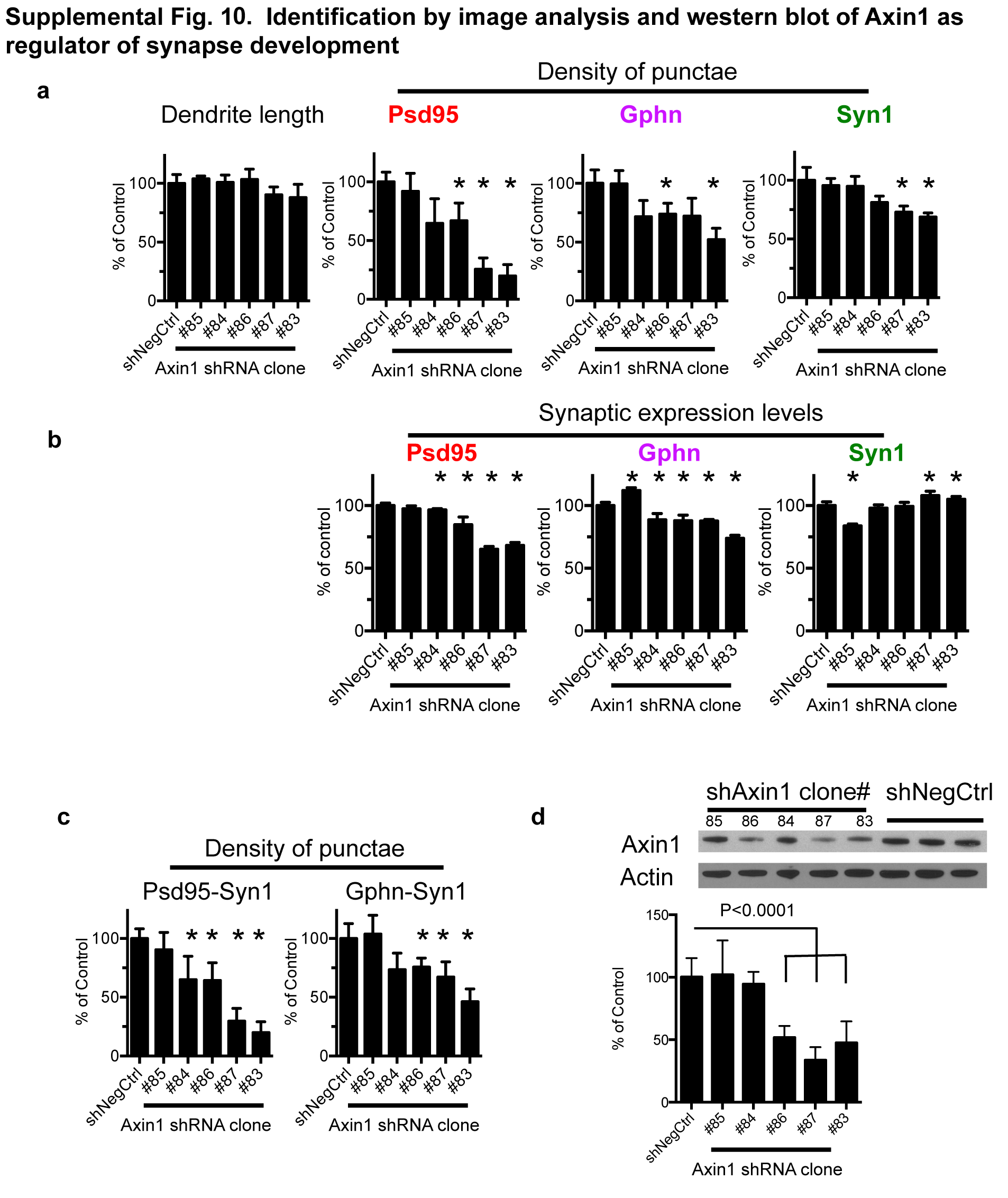

Supplement: Figure S10 — Identification by image analysis and western blot of Axin1 as regulator of synapse development. (A) The effect of all 5 shRNA reagents targeting Axin1 on synapse development and dendrite outgrowth was quantified using the synapse imaging algorithm (*p-values <0.05 or less (2-tailed t-tests, see table S2)) (B) shRNAs also reduce synaptic protein expression (mean and SD of primary screen results only) and (C) development of excitatory and inhibitory synapses. (D) shRNAs that reduced synapse development also reduced expression of Axin1 in DIV14 cortical mouse neuronal cultures confirming the on-target specificity of these RNAi. (A) and (D) present median and median absolute deviations of the primary screen and two secondary screens. Statistical analysis is presented in Table S2 and S3. (TIF) [file pone.0091744.s010.tif]

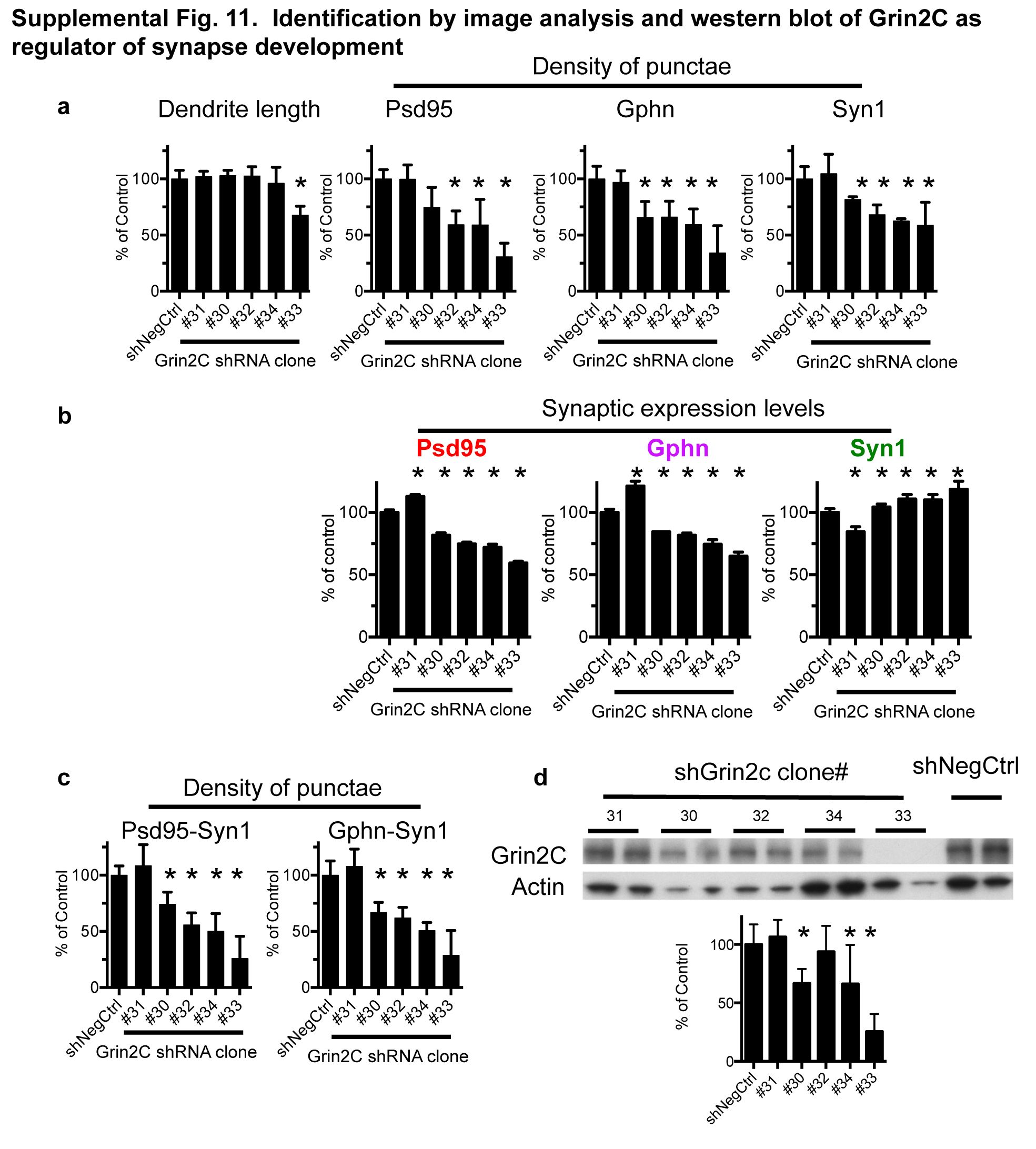

Supplement: Figure S11 — Identification by image analysis and western blot of Grin2C as regulator of synapse development. (A) The effect of all 5 shRNA reagents targeting Grin2C on synapse development and dendrite outgrowth was quantified using the synapse imaging algorithm (*p-values <0.05 or less (2-tailed t-tests, see table S2)) (B) shRNAs also reduce synaptic protein expression (mean and SD of primary screen results only) and (C) development of excitatory and inhibitory synapses. (D) shRNAs that reduced synapse development also reduced expression of Axin1 in DIV14 cortical mouse neuronal cultures confirming the on-target specificity of these RNAi. (A) and (D) present median and median absolute deviations of the primary screen and two secondary screens. Statistical analysis is presented in Table S2 and S3. (TIF) [file pone.0091744.s011.tif]

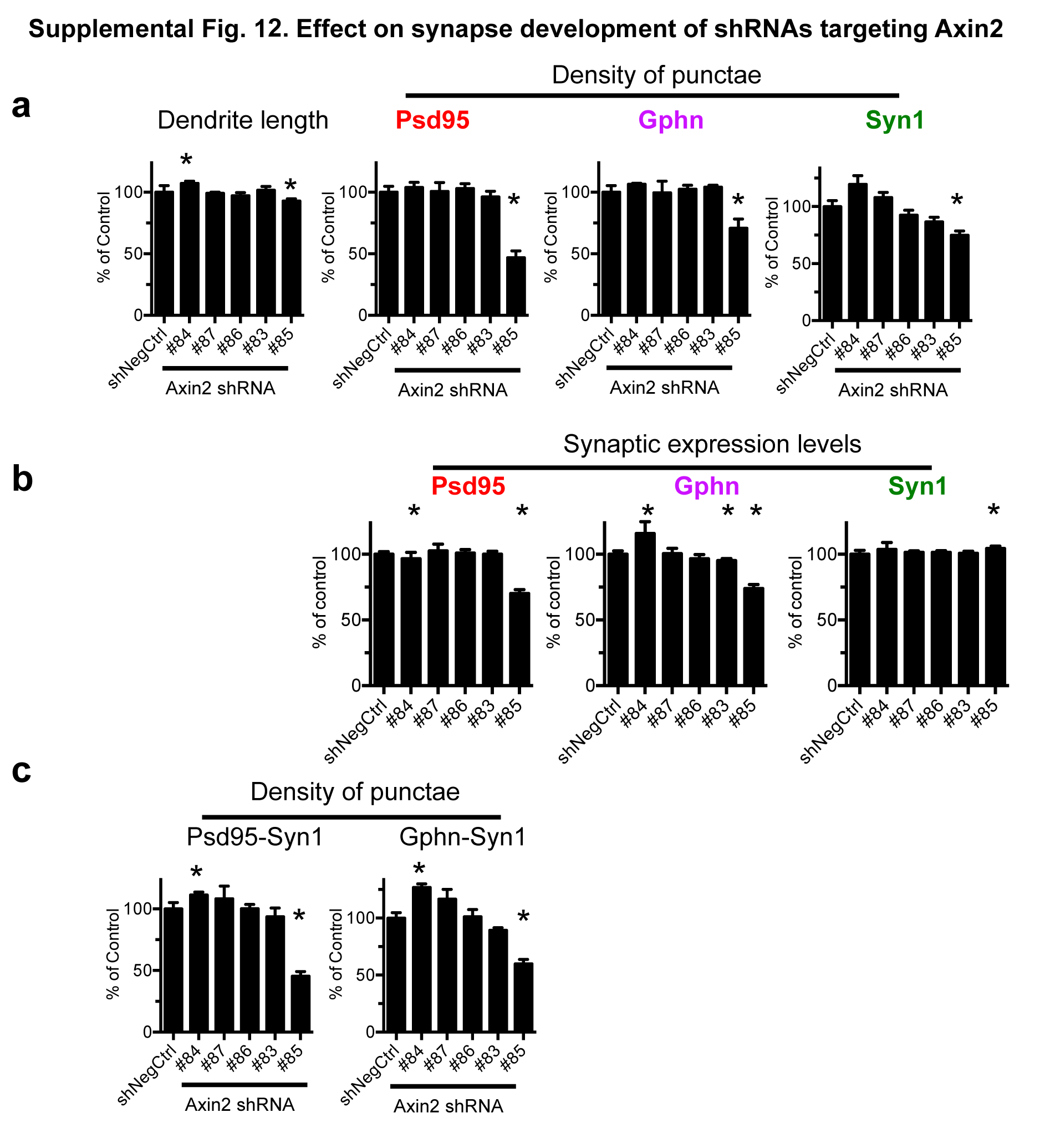

Supplement: Figure S12 — Effect on synapse development of shRNAs targeting Axin2. (A) The effect of all 5 shRNA reagents targeting Axin2 on synapse development and dendrite outgrowth was quantified using the synapse imaging algorithm (*p-values <0.05 or less (2-tailed t-tests, see table S2)) (B) shRNAs also reduce synaptic protein expression (mean and SD of primary screen results only) and (C) development of excitatory and inhibitory synapses. Data present the average data of the primary RNAi screen. Statistical analysis is presented in Table S2 and S3. (TIF) [file pone.0091744.s012.tif]

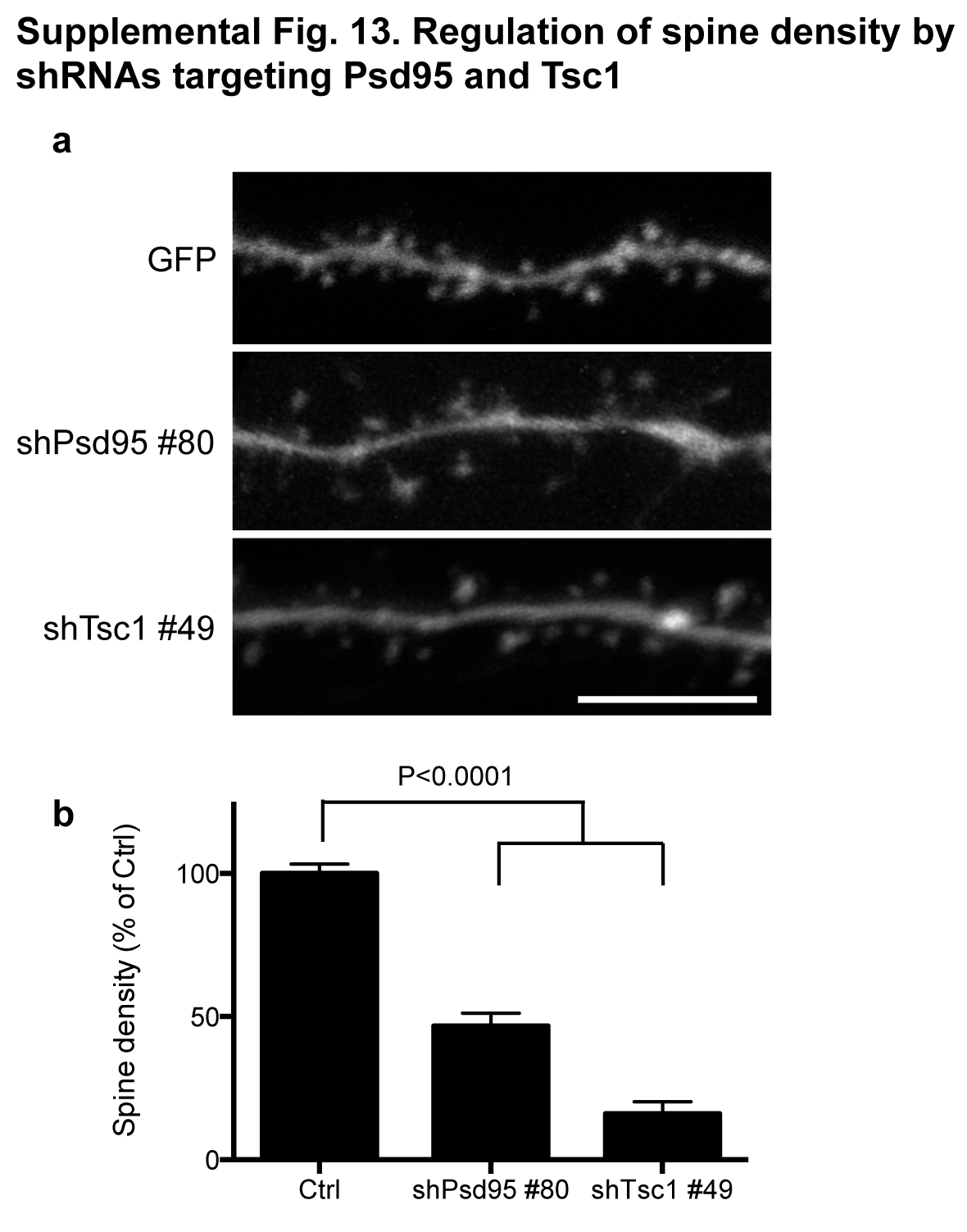

Supplement: Figure S13 — Regulation of spine density by shRNAs targeting Psd95 and Tsc1. Orthologous spine density assays experiments confirm that shRNA to Psd95 #80 and Tsc1 #49 reduce spine density qualitatively (A) and quantitatively (B) p<0.0001 (2-tailed T-test compared to GFP expressing vector) Scale bar denotes 10 μm. (TIF) [file pone.0091744.s013.tif]
